# Application of Sodium Polyacrylate Superabsorbent Polymer on Moisture Stability of Clay and Prediction of the Suction Potential of the Mixture

**DOI:** 10.3390/polym18182187

**Published:** 2026-09-08

**Authors:** Elahe Jafari, Jie Huang, Drew W. Johnson

**Affiliations:** 1 Group Delta Consultants, Inc., 32 Mauchly, Suite B, Irvine, CA 92618, USA; elahejafari87@gmail.com; 2 School of Civil and Environmental Engineering & Construction Management, University of Texas at San Antonio, San Antonio, TX 78249, USA; drew.johnson@utsa.edu

**Keywords:** superabsorbent polymer, clay, suction, soil–water characteristic curve

## Abstract

Superabsorbent polymers (SAPs), such as sodium polyacrylate (PAAS), are the neutralized form of poly (acrylic acid) and belong to a class of materials characterized by three-dimensional networks of flexible polymer chains with exceptional water absorption and retention capacities. Due to these properties, PAAS has been widely used in agriculture as a soil water conditioner. This study investigates the potential of PAAS as a soil stabilizer for infrastructure applications. Three suction measurement techniques, namely the axis translation method, osmotic technique, and vapor equilibrium method, were employed to determine the suction behavior of PAAS and soil–PAAS mixtures over a wide range of water contents and to develop their soil–water characteristic curves (SWCCs). The investigation covers the full suction spectrum, from near-complete dryness to full saturation. Experimental results show that the SWCC of PAAS exhibits the three characteristic zones commonly observed in soils: boundary, transition, and residual zones. However, when PAAS is mixed with soil, the boundary and transition zones disappear from the SWCCs of the soil–PAAS mixtures. This behavior is attributed to the suppression of PAAS suction capacity caused by soil confinement. Unlike agricultural applications, where SAP particles are relatively unconstrained, engineering applications typically involve highly compacted soils that restrict polymer expansion and water absorption. The study also evaluates the feasibility of predicting the suction behavior of soil–PAAS mixtures using numerical modeling techniques based on limited experimental datasets. Among the methods considered, Lagrange interpolation and K-nearest neighbors (KNN) produced prediction models with errors below 10%. In contrast, deep neural network models demonstrated lower predictive accuracy, primarily due to the limited size of the available dataset.

## 1. Introduction

Failures in earthen structures and foundations are often associated with changes in water content, as the mechanical behavior of soils is highly dependent on moisture conditions [1,2]. Soil with more clay content is more prone to shrinkage and swelling upon wetting and drying [3]. Clayey soil also tends to partially lose shear strength and be significantly softened due to the increase in water content. To solve the issue, various ground improvement methods, particularly through mechanical or chemical modifications/stabilizations, are often used when excessive water is present in clayey soil [1,4,5].

Mechanical stabilization relies on physical techniques to improve soil properties. Common mechanical stabilization methods include over-excavation and replacement, soil blending, dynamic compaction, vibro-compaction, preloading, and consolidation. These approaches enhance soil performance by increasing soil density or incorporating materials that are less sensitive to moisture variations, thereby reducing the adverse effects of water-content fluctuations on soil shear strength and stiffness, and in some cases improving these properties [1,6]. However, the application of mechanical stabilization methods is often constrained by their high cost, lengthy construction time, site disturbance, and suitability only for localized treatment areas [5].

Chemical stabilization is commonly achieved through the addition of traditional stabilizing agents such as lime, cement, and fly ash. These materials promote cementation within the soil matrix, thereby enhancing soil shear strength and stiffness while simultaneously reducing water migration by blocking flow pathways [7,8,9]. Although traditional chemical stabilization techniques have been successfully applied to a wide range of soils, they may pose environmental concerns, including irreversibility, high carbon emissions, and decreased soil fertility. Furthermore, the relatively long curing periods required for strength development represent an additional limitation of these methods [10,11].

Non-traditional stabilizers, such as potassium compounds, polymers, ammonium chloride, sulphonated oils, fibers and enzymes, are introduced in the literature as a low-cost alternative for stabilization [5,12,13]. Environmentally friendly polymers have also attracted considerable attention in recent years [14]. Having excellent tensile and flexural strengths, polymers coat soil particles and create strong bonds promoting adhesion but they are most compatible with granular soils [12]. These non-traditional soil stabilizers may have better performance than traditional stabilizers but also share some similar disadvantages such as changing the chemical environment in the soil or preventing vegetation.

In addition to stabilization techniques, site water content can be managed with minimal disturbance by either preventing water from entering or leaving the soil mass or by rapidly removing infiltrating water. Restricting water ingress is commonly achieved through the installation of hydraulic barriers, such as cutoff walls, clay liners, or low-permeability geomembranes, which may be placed in either vertical or horizontal configurations. These barriers reduce water migration into the soil and help maintain more stable moisture conditions within the treated area [1,6,15]. Electro-osmosis removes water by inducing electric currents, which is suitable for clay with very low permeability and likely will result in soil densification [16]. Drainage and filtration systems facilitate water movement, allowing excess water to be continuously removed while simultaneously retaining soil particles and preserving soil integrity [1,2]. Although the underlying principle is straightforward, implementation is often challenging due to the inherent variability of soil permeability and the complexity of predicting three-dimensional groundwater flow patterns. Geotextiles have increasingly replaced conventional drainage systems because of their cost-effectiveness, ease of installation, and ability to provide filtration while minimizing particle migration and material segregation under hydraulic gradients [15,16]. Conventional geotextile drainage systems are generally most effective under saturated soil conditions. In unsaturated soils, however, capillary-held water may not be readily removed by traditional drainage methods. To address this limitation, enhanced lateral drainage (ELD) geotextiles, also known as wicking geotextiles, have been developed to promote moisture removal under unsaturated conditions. These geotextiles contain deeply grooved fibers with a unique cross-sectional geometry that functions as a network of capillary tubes, generating capillary forces that draw water from the surrounding soil and transport it away from the treated zone [17]. Using a physical model test, Wang et al. [18] evaluated wicking geotextiles’ moisture reduction capabilities for roadways and showed that wicking geotextiles are effective in draining water out of soil at moistures close to optimum water content. Long-term performance of ELD geotextiles may be impacted by clogging and splices. In a series of laboratory experiments performed by Lin and Zhang [19], they found that splices reduce the efficiency of wicking geotextiles but clogging is not a major concern for soils containing less than 14% fines.

Although several methods are available for controlling soil moisture, many are constrained by site conditions, require significant construction effort, involve high costs, or may pose environmental concerns. Consequently, there is a need for durable, cost-effective, environmentally sustainable, and vegetation-friendly solutions that can improve soil performance under varying moisture conditions. One promising approach is the incorporation of superabsorbent polymers (SAPs) into soils. SAPs are capable of absorbing and retaining large quantities of water and can therefore help regulate soil moisture content. Under wet conditions, SAP particles absorb excess water and reduce moisture fluctuations, whereas under dry conditions, they gradually release stored water back into the surrounding soil. By functioning as a moisture buffer, SAPs can mitigate variations in soil water content and improve moisture stability, with water absorption capacities reaching up to 100 times their own mass.

SAPs have been extensively investigated for their water retention capabilities, primarily in agricultural applications aimed at improving water availability to plants [20]. These studies are primarily focused on the impact of SAP on hydraulic properties of sandy soil and have found that the hydraulic conductivity of SAP–soil mixtures decreases as the ratio of SAP increases [21,22,23,24]. The effect of SAP on the soil–water characteristic curve (SWCC) and water retention behavior of coarse-grained soils have been studied especially for agricultural applications, where SAP is used as a soil conditioner [20,21,25]. The suction was mostly applied using the pressure plate apparatus in a range between 0 and 1500 kPa. The results of soil–water retention curves revealed that the unamended soil retained less water at any matric potential compared to the SAP–soil mixtures [26,27]. The efficiency of the SAP in water absorption from and release back to soil is highly influenced by soil properties as well as SAP type and concentration [28]. With an increase in SAP concentration, the water retained by SAP–soil mixtures also increases [20,29,30]. Salinity of soil has been found to reduce the water-absorbing capacity of SAPs. This decrease in absorbency is primarily due to the presence of cations in saline solutions, particularly multivalent ones, which displace water molecules from the polarized sites of the SAPs’ molecular network, resulting in less water being absorbed [21,30,31,32,33].

Several studies have examined the influence of SAPs on fine-grained soils and have reported that the increase in water content at saturation resulting from SAP addition is generally more pronounced in sandy soils than in clayey soils [28]. Narjary et al. [24] found that by applying 0.7% SAP to soil, the moisture content at saturation of SAP–soil mixtures increases by 21% in black clay, 40% in sand, and 24% in alluvial and red sandy loam soils. Abedi-Koupai et al. [34] showed that the saturated water content of 0.8% SAP–soil mixtures is 2.2 and 1.5 times that of untreated soil for sandy loam soil and clay soil, respectively. Geotechnical applications of SAPs require a thorough understanding of the effects of surcharge loading on their water absorption behavior. Lejcus et al. [35] investigated the absorbency of two SAPs, i.e., cross-linked copolymer of acrylamide and potassium acrylate, under loads varying between 0.5 and 3.8 kPa, and showed that the absorption capacity of SAP significantly decreases under loads. Misiewicz et al. [36] investigated the water absorption capacity of the same SAPs when incorporated into coarse sand and sandy loam under applied loading conditions. Their findings demonstrated that the absorbency of the SAP–soil mixtures decreased as the applied load increased, highlighting the restrictive effect of confinement on SAP swelling and water uptake.

Although SAPs have been widely utilized in agricultural applications, research on their behavior in soils remains relatively limited and has largely focused on their role as soil conditioners for enhancing plant growth and water availability, with little attention given to their influence on soil engineering properties. Consequently, there is a need to reassess and expand existing knowledge of SAPs within the context of civil engineering applications. The present study investigates the effects of SAPs on soil–water interactions through the soil–water characteristic curve (SWCC), which provides a fundamental framework for understanding unsaturated soil behavior. The SWCC serves as the basis for evaluating the influence of SAPs on a range of geotechnical properties and processes, including volume change, shear strength, hydraulic conductivity, adsorption, diffusion, and consolidation behavior [3,37]. In addition, analytical models were developed in this study to predict SWCCs for soil with different dosages of SAP. The accuracy of the models was then verified by comparing the result of the model for soil with 1% SAP with the established SWCC of the same sample. Moreover, it is important to recognize that the swelling and water absorption behavior of SAPs differs significantly when they are incorporated into soil compared with their free-swelling state. Interactions between SAP particles and soil grains, together with the effects of soil structure and pore-size distribution, can alter the absorption capacity of the polymer by restricting its expansion and water uptake. To better understand these mechanisms, this study compares the soil–water characteristic curves (SWCCs) of standalone SAP and SAP–soil mixtures, thereby evaluating the influence of soil confinement on SAP hydraulic behavior.

## 2. Materials and Methods

### 2.1. Materials

The soil used in this study was clay with a maximum dry unit weight of 17.5 kN/m^3^, liquid limit of 34%, plastic limit of 15%, plasticity index of 19%, and shrinkage limit of 12%, which were obtained according to ASTM standards [38,39] and are summarized in Table 1.

**Figure 1:**
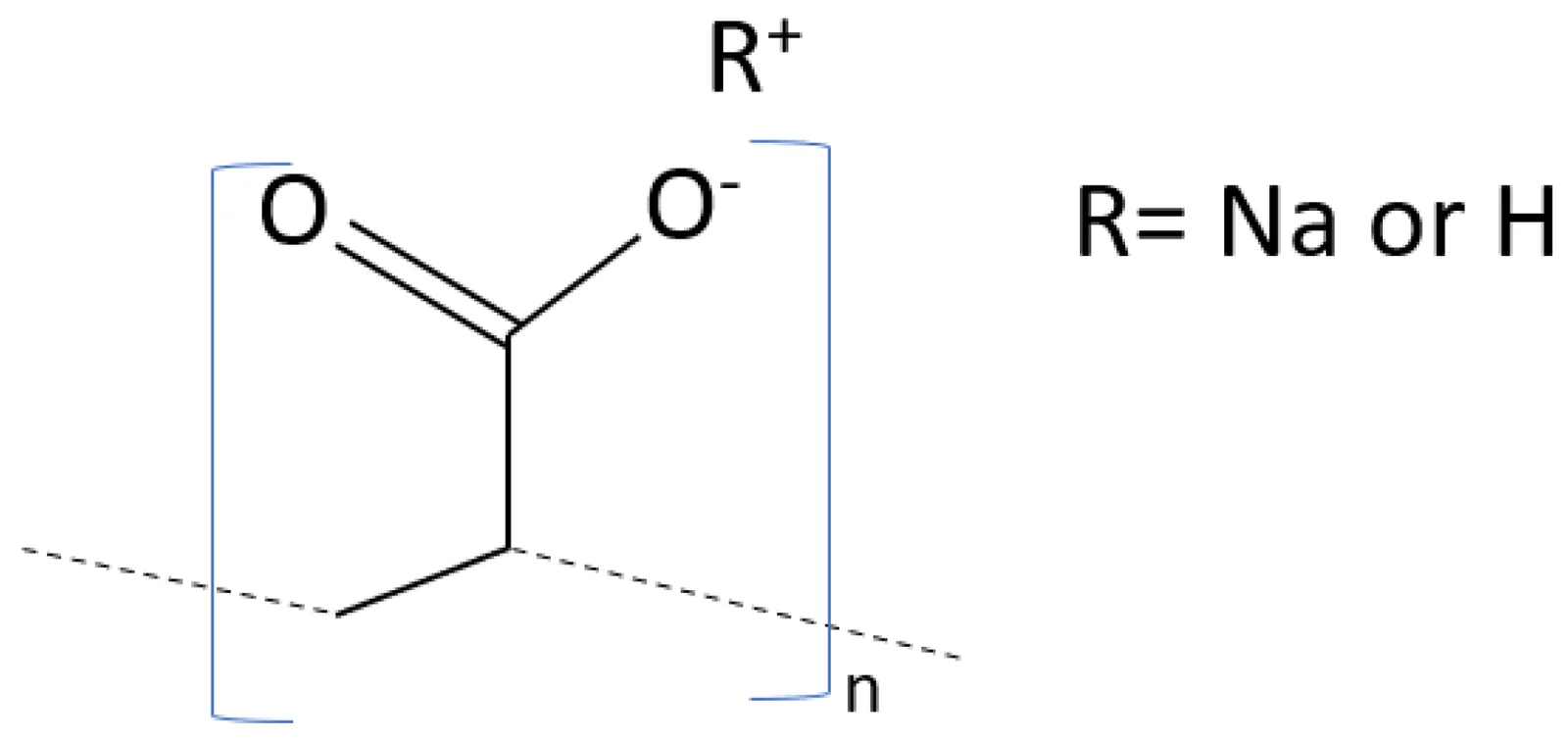
Chemical compound of sodium polyacrylate [40].

**Figure 2:**
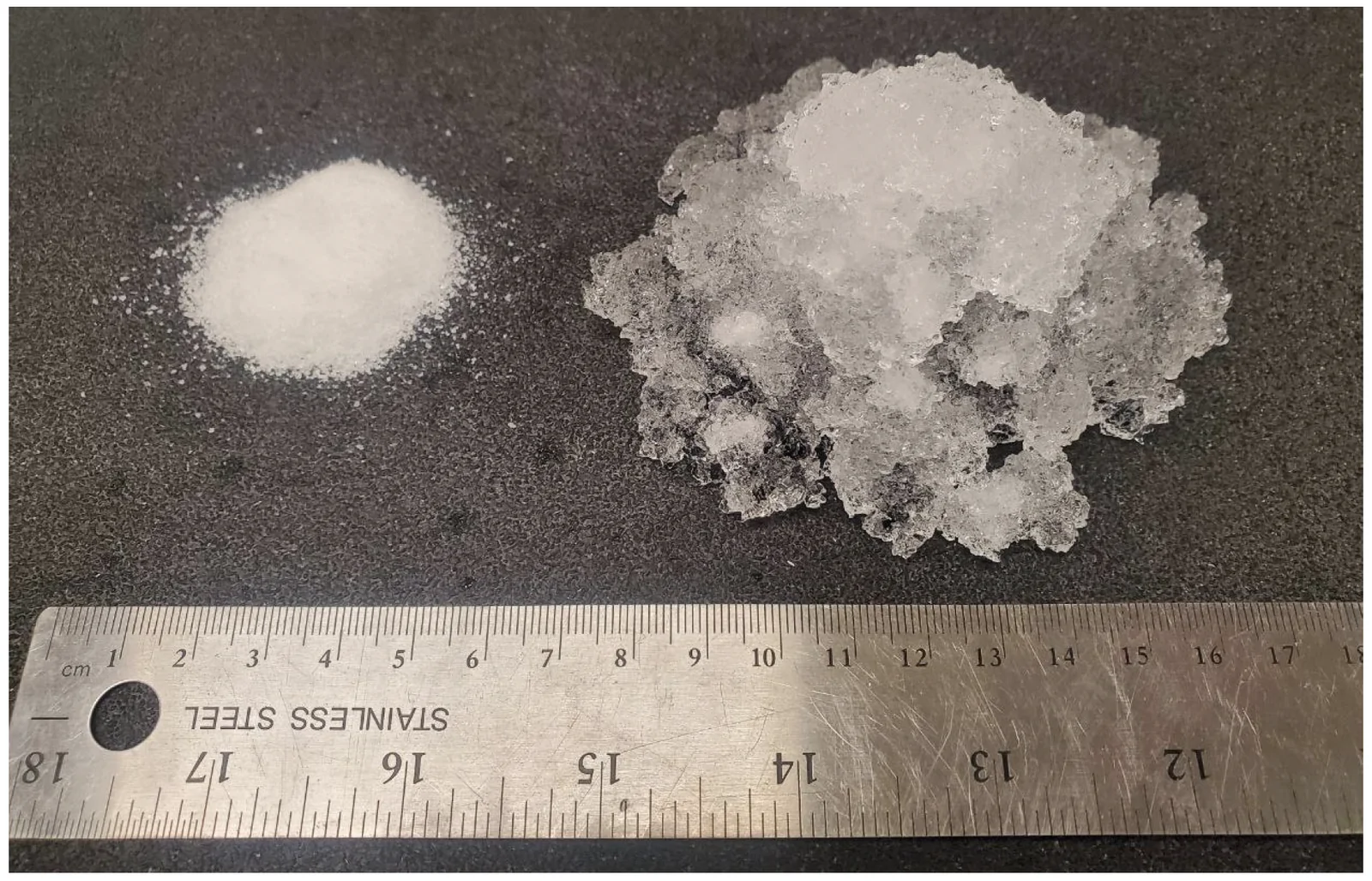
Sodium polyacrylate size and form in dry state (**left**) versus water swollen gel (**right**).

**Figure 3:**
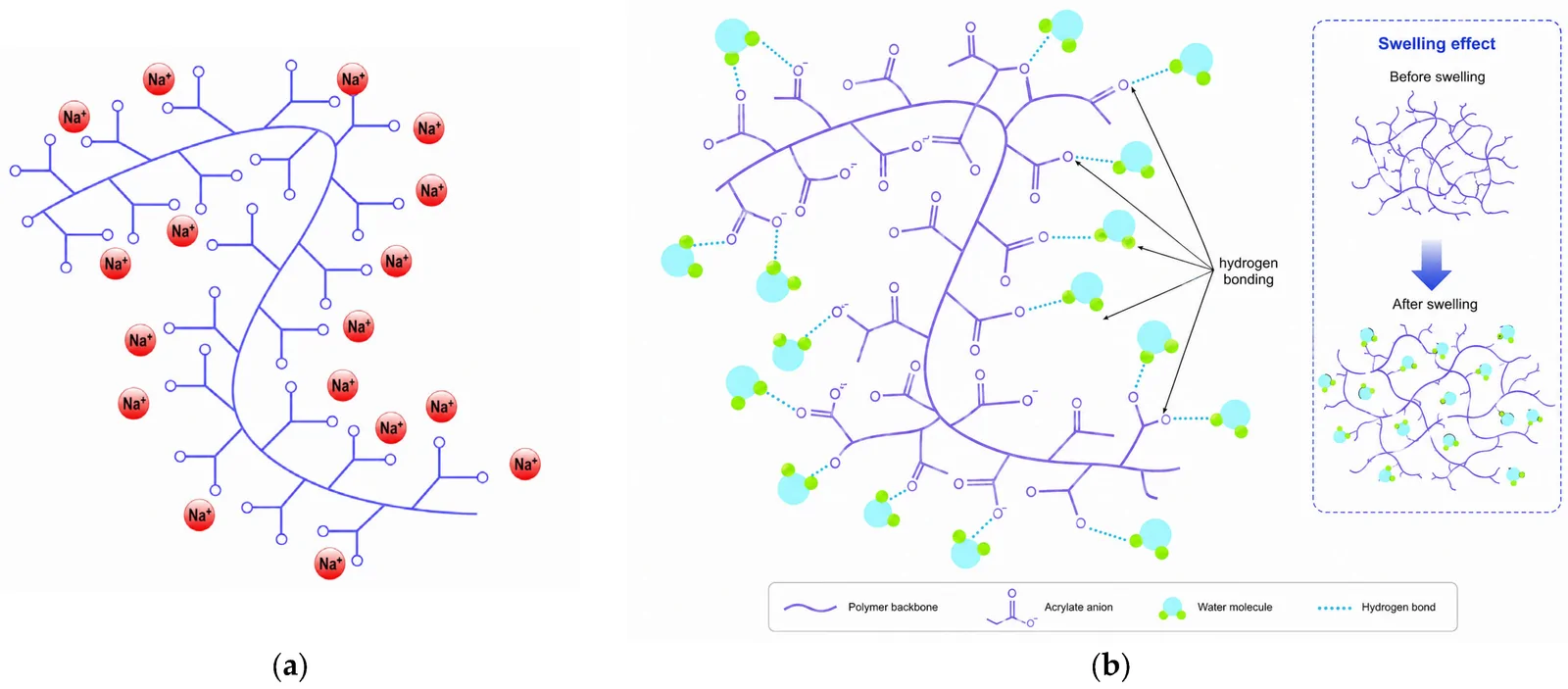
The structure of sodium polyacrylate in (**a**) dry form versus (**b**) with added water [41].

The SAP used in this study is sodium polyacrylate (PAAS) that is the neutralized form of poly (acrylic acid) [40]. Figure 1 shows the chemical compound of PAAS. The used PAAS was purchased from Sigma-Aldrich (St. Louis, MO, USA) in the dry, white powder form. The PAAS is cross-linked and hydrophilic and does not dissolve. The product is a powder in its dry form with particle size between 90 and 850 μm and density of 0.54 g/mL at 25 °C [40], as shown in Figure 2. When sodium polyacrylate is dry, in each polyacrylate chain, the negatively charged carbonyl groups of the polymer backbone hold the positively charged sodium ions close together as shown in Figure 3a. Figure 3b shows how adding water alters the interaction and unravels the tightly organized chains of the polymer by replacing the sodium ions with positively charged hydrogen ions [41]. As a result, upon adding water the polymer network swells dramatically and forms a swollen gel, as shown in Figure 2. Because of the cross-links between network chains, PAAS is resistant to dissolution in water [42].

The tea bag method was utilized to assess the maximum absorption capacity of sodium polyacrylate for three samples. The test outcomes indicated that the PAAS could absorb nearly 400 times its original mass within 24 h. Additionally, within just 30 min, it can achieve 90% of its peak absorbency [43]. For this study, to form PAAS–soil mixtures, 0.5%, 1%, or 2% of sodium polyacrylate in the form of dry powder was added to dry soil.

### 2.2. Experiments

A general consensus has emerged within the geotechnical engineering community regarding the laboratory methods used to determine and interpret soil–water characteristic curves (SWCCs) [44]. Numerous studies have evaluated the available testing techniques for establishing SWCCs [45,46]. These methods differ in terms of complexity, accuracy, repeatability, applicable suction range, testing duration, and cost. For example, the vapor equilibrium technique is relatively economical but requires extended testing periods [47]. In contrast, the pressure plate method enables rapid testing of multiple specimens simultaneously; however, it is costly and applicable only within a limited suction range [48]. Consequently, most previous studies have focused on relatively narrow suction ranges relevant to specific engineering applications. To address this limitation and meet broader engineering needs, the present study combined the axis translation, osmotic, and vapor equilibrium techniques to develop complete SWCCs for clay and PAAS–soil mixtures over a suction range extending from near-zero values to greater than 10 MPa. The following section describes these testing methods and the experimental procedures adopted in this study.

#### 2.2.1. Axis Translation Technique

**Figure 4:**
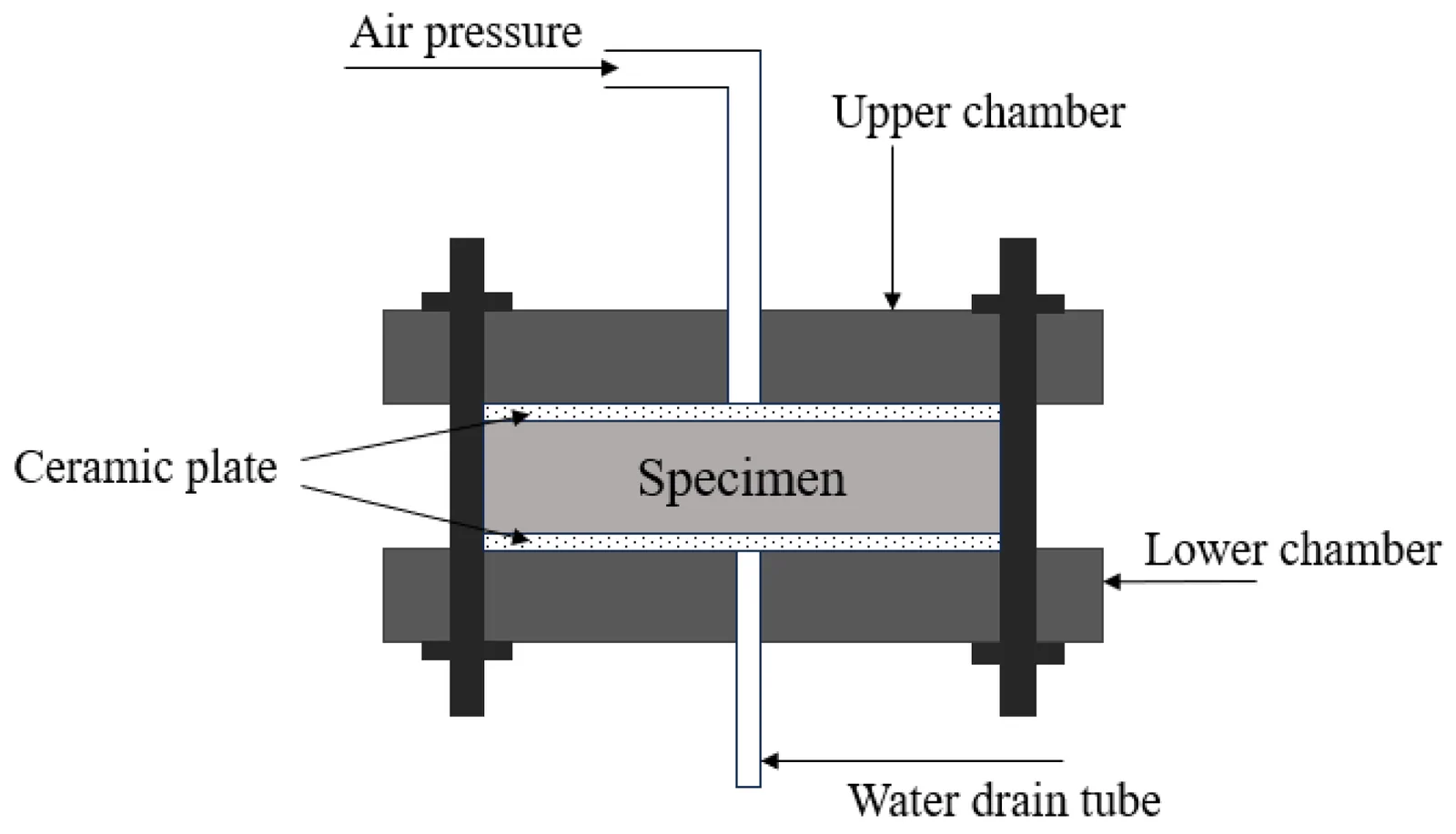
Schematic of pressure chamber with ceramic porous disk [52].

The axis translation technique (ATT) is a soil suction measuring method used to measure or control matric suction in unsaturated soils by applying elevated pore-air pressure. During the test, matric suction can be directly controlled by increasing air pressure while maintaining pore water pressure equal to atmospheric pressure. This could be done in a pressure plate or a Tempe cell [48]. Figure 4 shows the schematic of a Tempe cell pressure chamber with ceramic disk. In this method, high-air-entry ceramic disks act as a membrane between air and water for direct measurements of matric suction. However, these disks possess two notable limitations: an extremely low permeability coefficient and gradual air diffusion through the water phase [44]. The low permeability coefficient constrains the rate of pore pressure response and impedes the release of water from the soil. Additionally, the high-air-entry disk sets a ceiling on the maximum permeability coefficient that can be measured in a laboratory. Finally, the air-entry value of the ceramic disk restricts the maximum matric suction that can be applied [44,49]. The maximum matric suction that can be maintained across the disk surface (air-entry value) depends on the pore size of the disk. Most pressure plates are limited to 1500 kPa air-entry value, and suctions above that must be applied with other methods [50]. Richards [51] used a pressure membrane extraction apparatus for obtaining curves up to 1600 kPa.

**Figure 5:**
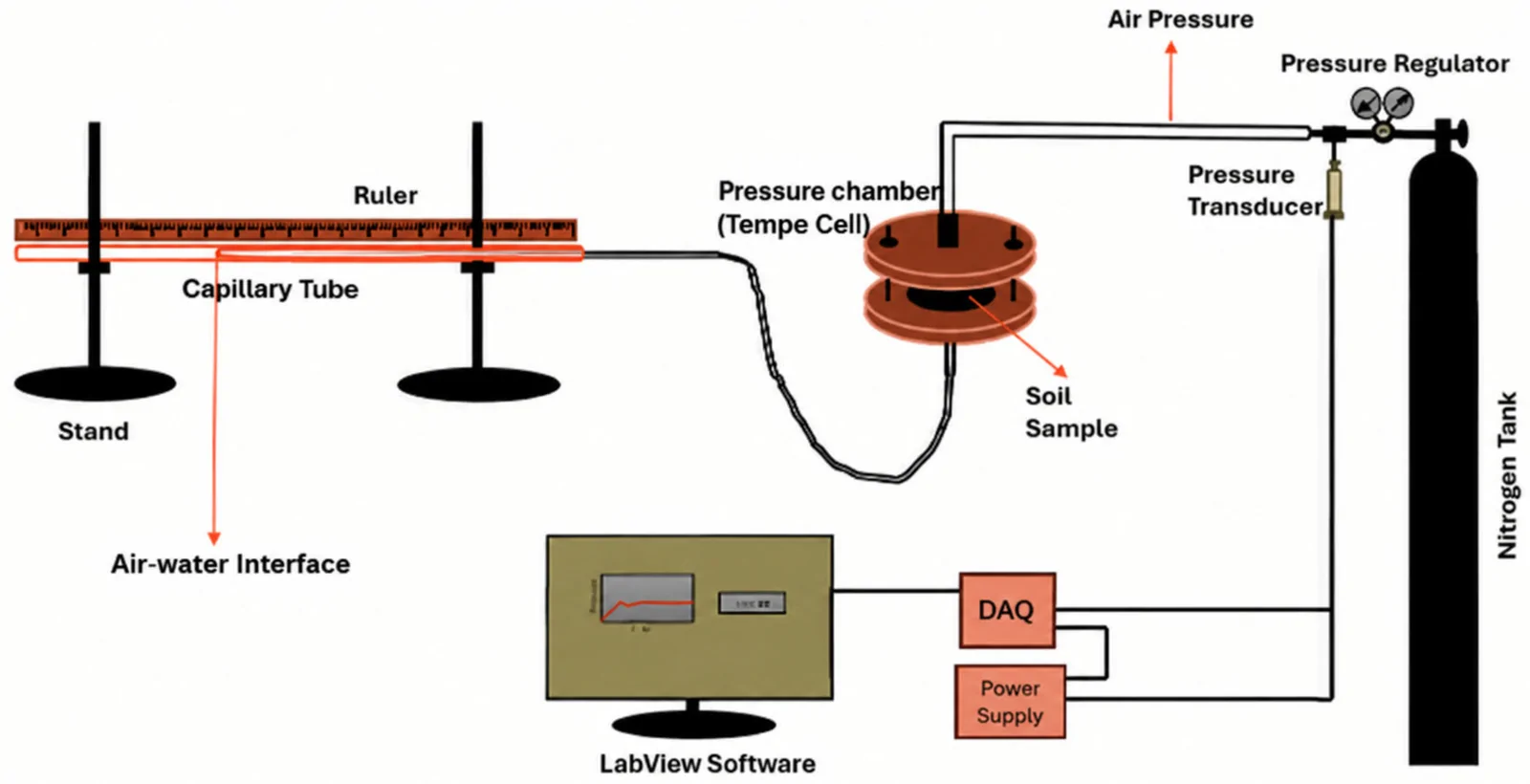
The schematic of the axis translation method with Tempe cell.

The test setup of the ATT method for this study is shown in Figure 5. In this study, a Tempe cell was used to carry out the axis translation method for the suction range of 0 to 100 kPa. In the test, the saturated sample was placed inside a Tempe cell pressure chamber with a porous ceramic disk on upper and lower surfaces, acting as a membrane between air and water. The gas pressure forced the excess water to be expelled out through the microscopic pores of the disk. Since the porous disk was completely saturated and the diameter of the pores was very small, the disk allowed the water to pass but prevented the passage of air. The volume of expelled water is measured by the change in the air–water interface location within a capillary tube attached to the bottom of the Tempe cell. In order to apply gas pressure to the test specimen, a nitrogen tank was used, which had a maximum pressure of 20 MPa. To monitor the gas pressure applied to the specimen, a pressure transducer was attached to the system with the data acquisition system (DAQ), controlled by LabView. The real-time data can be reviewed on LabView, allowing timely pressure adjustments if needed.

The water content of the sample after equilibrium can be obtained either volumetrically or gravimetrically. For the clay sample, since the change in the volume of sample was negligible, the volumetric water content at any pressure was calculated based on the volume of expelled water and the saturation water content of the sample as shown in Equation (1).

(1) $$\theta_{i}=\theta_{i-1}-\left(x_{i}-x_{i-1}\right)\frac{F}{V}$$

where $\theta_{i-1}$ is the volumetric water content at the previous suction and $\theta_{o}$ is the water content at saturation; $x_{i}$ is the location of the air–water interface in the capillary tube after equilibrium has been established at the ith suction; $x_{i-1}$ is the location of the air–water interface in capillary after equilibrium was established for the previous increment in suction; F is the scale factor for the capillary tube, which converts the volume to displacement of the air–water interface; and V is the volume of the sample.

For the PAAS–soil mixtures, the volume changes are significant, and the gravimetric water content is measured by the oven drying method after removing the sample from the Tempe cell with the calculation based on Equation (2).

(2) $$\omega =\frac{w_{wet}-w_{dry}}{w_{dry}}\times 100$$

where W_wet_ is the weight of sample after removing from the Tempe cell and W_dry_ is the weight of sample after oven drying for two days. For the suction test, the time each specimen requires to reach equilibrium raises with the increase in pressure or PAAS dosage. For instance, for the specimen under 10 kPa pressure, the equilibrium time was 24 h, while the PAAS specimen at 30 kPa pressure required more than one week to reach equilibrium.

#### 2.2.2. Vapor Equilibrium Technique

**Figure 6:**
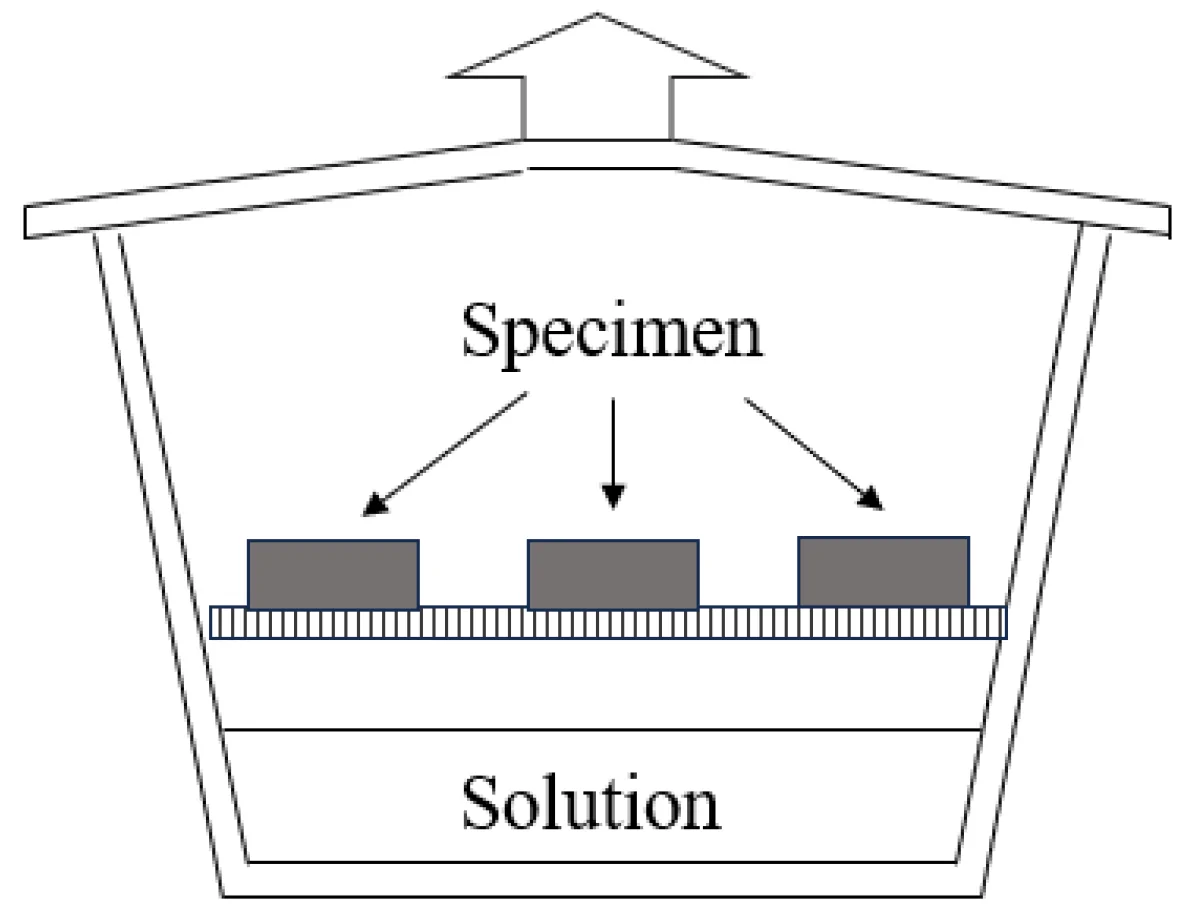
Soil specimen in constant suction environment in vapor equilibrium technique, redrawn from [47].

In this method, a constant suction environment is created in sealed containers using salt solutions that generates a specific relative humidity [47]. During the test, the soil is placed in the vapor environment above a salt solution in a desiccator, as shown in Figure 6. The salt solution has a high affinity for water and is used to absorb moisture. Therefore, suction is applied to the soil sample by the water exchange between the specimen and the vapor in the air of the desiccator. Over time, water in the soil will evaporate or be absorbed to reach equilibrium with the surrounding vapor pressure, thereby changing the soil’s water content. While the vapor equilibrium technique provides an economical and straightforward means of controlling total suction, attainment of equilibrium can be time-consuming because vapor transport within the system is inherently slow [47,53]. This method was first used to measure soil suction in the 1980s [54] and has become a mature method with conversions between relative humidity and concentrations of various commonly used salt solutions widely available in the literature [55,56].

The vapor equilibrium technique is a suitable method to measure high suctions since dry environments can be easily created by increasing the concentration of the salt solution. Saiyouri et al. [57] used salt solutions to impose relative humidity up to 107 MPa to soil samples inside a desiccator. Due to the difficulty of controlling high relative humidity values that require small quantities of solute, a lower limit of 3 MPa is suggested by Romero et al. [53] as the total suction lower limit. Delage et al. [54] reported that a 1% uncertainty in relative humidity corresponds to an absolute suction uncertainty of approximately 1.38 MPa. Such an error is generally acceptable only at high suction levels, further confirming that the vapor equilibrium technique is best suited for measuring high suctions.

In this study, three desiccators are used to impose three different suctions to the samples by creating different relative humidities. The bottom half of each desiccator is filled with CaCl_2_ solutions at different concentrations that is calculated based on required relative humidities according to the relation presented by Bulut et al. [56] shown as Equation (3).

(3) h=νRTmϕ

where h is total suction; ν is the number of ions from one molecule of salt (i.e., ν is 3 for CaCl_2_); R is the universal gas constant (8.31432 Pa·m^3^/mol·K); T is the absolute temperature; ϕ is the osmotic coefficient used from the table presented by Bulut et al. [56]; and m is molality.

**Figure 7:**
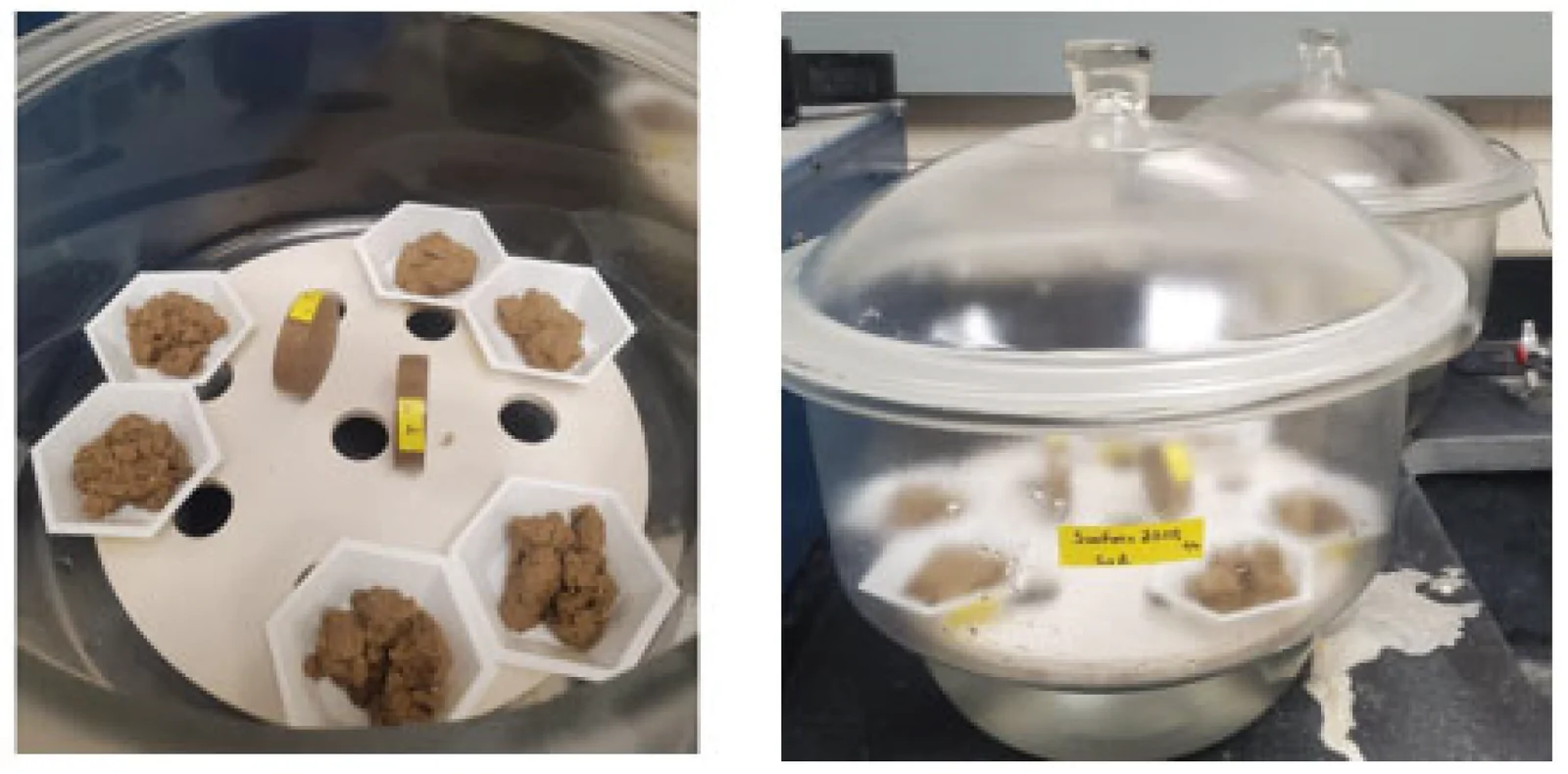
Saturated samples of soil and PAAS–soil mixtures inside the desiccator.

During the test, the saturated samples of soil and PAAS–soil mixtures at different PAAS ratios are placed in the desiccators sitting on top of the CaCl_2_ solution as shown in Figure 7. Two desiccators with high concentrations of salt created suctions of 13 MPa and 20 MPa were used to complete the far end of the SWCC for PAAS–soil mixtures, while the 3rd desiccator was used at a lower suction of 67.5 kPa to allow a comparison with the results from the other two methods used in this study. The weight of each sample was monitored throughout the test until it became steady, which indicated an equilibrium. The samples are taken out of the desiccator once a month for a weight check earlier in the experiment and then every week as the test progresses. The soil sample reached equilibrium in 65 days, while the soil–PAAS mixtures required more than 160 days to reach equilibrium. The gravimetric water content of each sample was measured after equilibrium by oven drying.

#### 2.2.3. Osmotic Technique

**Figure 8:**
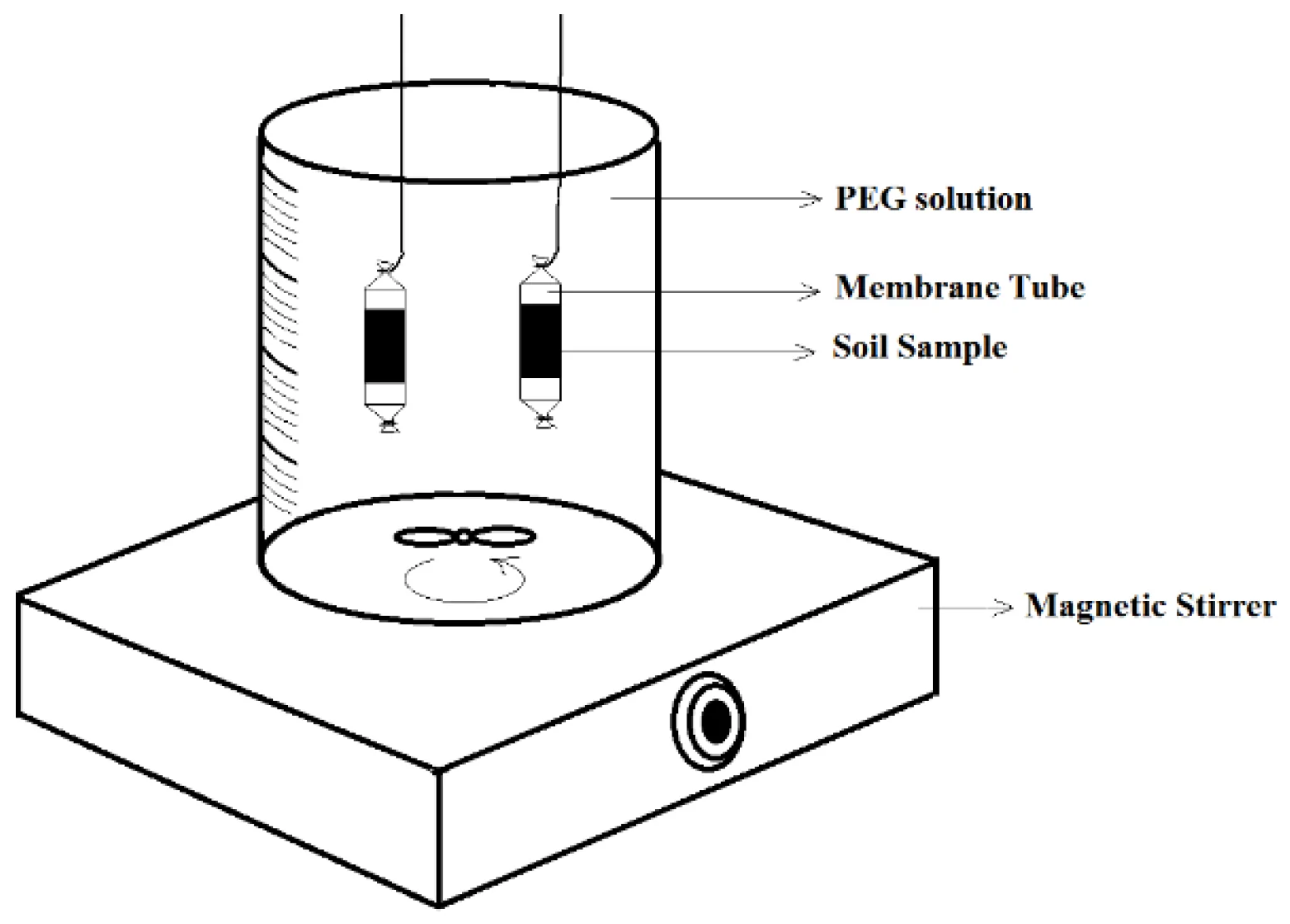
Soil samples wrapped with membrane tube immersed in PEG solution in osmotic technique.

The osmotic technique utilizes osmotic pressure to measure the matric suction of a soil, which in general is simple, safe, and inexpensive [47,58]. In this technique, water exchanges between soil specimen and a solution of polyethylene glycol (PEG) through a cellulose semi-permeable membrane under an osmotic suction [59]. PEG is a synthetic polymer that has a high affinity for water molecules due to its hydrophilicity and non-polar backbone [60]. The osmotic pressure of a PEG solution is the pressure required to prevent water from flowing across a semi-permeable membrane and depends on its concentration of the solution [61]. The use of the membrane ensures that only water molecules can pass through but not the PEG molecules [61]. This helps to maintain the integrity of the soil specimen and prevents the PEG from contaminating the soil specimen. The suction potential is defined as the difference in water potential between the soil specimen and the PEG solution, which is directly related to the osmotic pressure of the solution. Therefore, a higher concentration of PEG results in a higher suction potential, which allows the soil specimen to be subjected to higher matric suctions [58]. Since water exchange occurs in the liquid state, equilibrium occurs much faster than using the vapor equilibrium technique. Figure 8 shows the schematic of this technique for this study. Williams and Shaykewich [62] summarized relationships between solution concentration and osmotic potential from numerous sources for two PEG molecular weights of 6000 and 20,000. They also showed that at any of the matric potentials, no significant difference existed between the results of osmotic technique and the axis translation method. Delage et al. [54] extended the range of available suctions applied by the osmotic technique up to 10 MPa and in addition, using the experimental results collected by William and Shaykewich [62], they suggested a parabolic relation between PEG concentration and suction when suctions were no greater than 4 MPa, shown as Equation (4). Most of the studies indicated that the osmotic technique could be used for medium suction range, i.e., 50 kPa to 4 MPa, making it perfect to fill the measuring gap between the axis translation technique and the vapor equilibrium technique to generate a SWCC covering the whole range of suction.

(4) $$S=11c^{2}$$

where S is the suction expressed in MPa and c is the concentration expressed in g PEG/g water.

**Figure 9:**
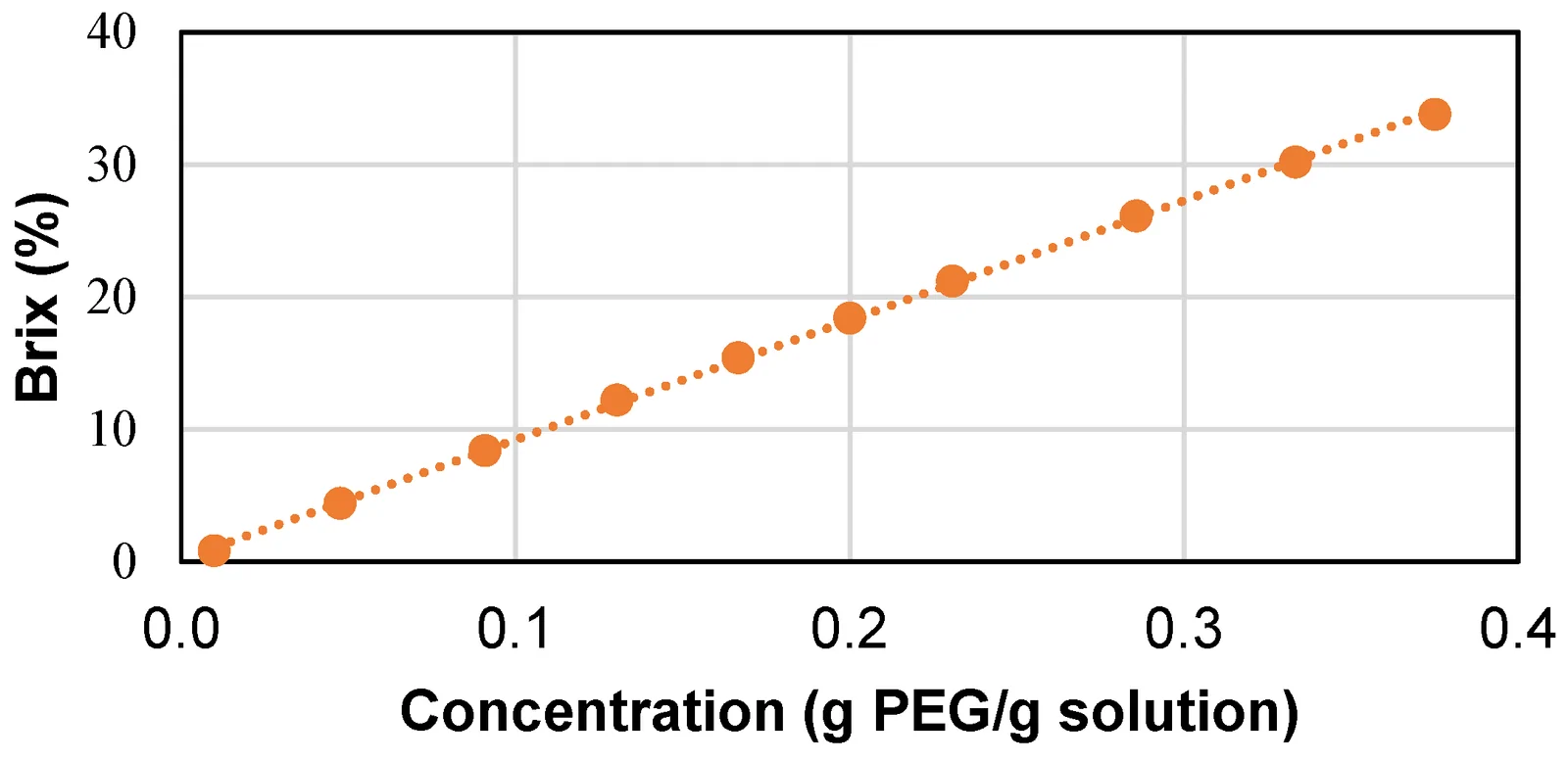
Calibration of refractive index of PEG solutions.

In this study, PEG with molecular weight of 20,000 was used to make different solutions with specific suction potentials varying from 50 kPa to 4 MPa based on the concentration of the solution. For each desired suction, the concentration of the PEG solution was calculated based on Equation (4). As the specimens were initially saturated, water migrated from the samples during the equilibration process, causing a slight decrease in the solution concentration at the end of each test. In order to prevent significant concentration fluctuation induced by small changes in water, a large quantity of PEG solutions was used for each test. In addition, the concentration of each solution was measured by a refractometer before and after each test to ensure the fluctuation of concentration was not beyond the acceptable range. A Reichert AE200 digital refractometer (Reichert, Depew, NY, USA) was employed in this experiment to check PEG concentration, which measures the refractive index, % solids as Brix, with an accuracy of ±0.1% Brix. The calibration of the refractometer Brix value versus concentration is shown in Figure 9, which is in complete agreement with the calibration results for the Brix value presented by Delage [54].

**Figure 10:**
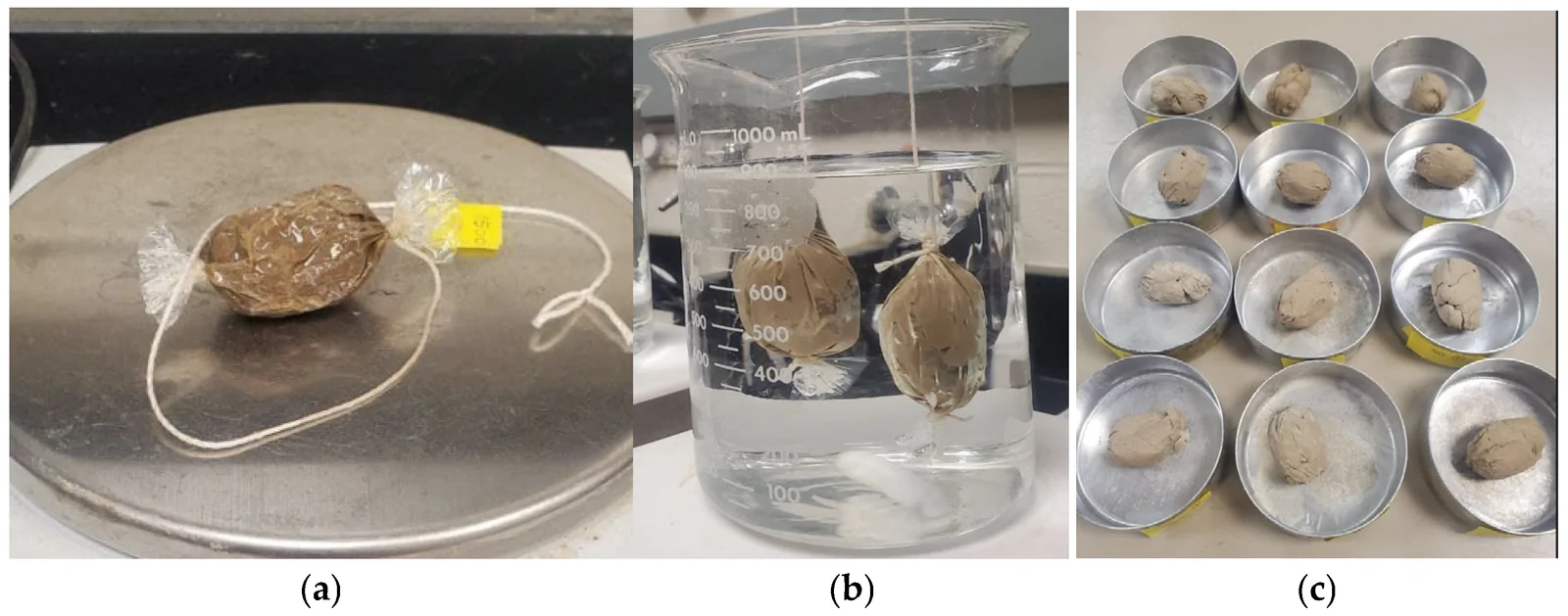
Osmotic technique procedure; (**a**): prepared sample wrapped with semi-permeable membrane; (**b**): prepared samples immersed in PEG solution; (**c**): oven-dried samples.

**Figure 11:**
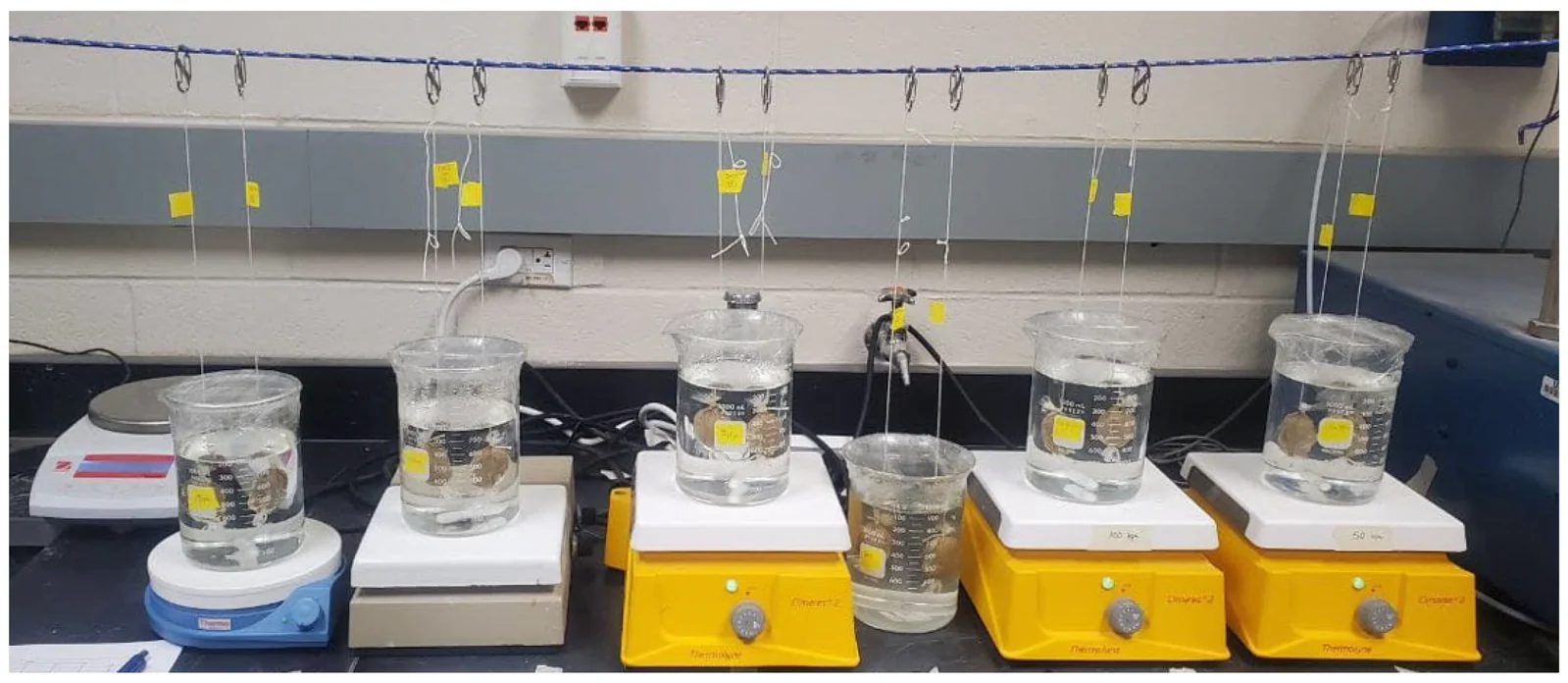
Soil samples under 6 different suctions in osmotic technique.

A SpectraPor membrane tubing (Repligen, Waltham, MA, USA) with molecular weight cutoff of 12–14 kD was used to wrap each sample and separate the soil and PEG solution as shown in Figure 10a. The samples of soil and PAAS–soil mixtures were immersed in the solution as shown in Figure 10b. The PEG solution was stirred continuously by using a magnetic stirrer to reduce the equilibrium time. The samples were taken out of the solution every day and weighed until equilibrium was reached for the water exchange between the sample and the solution. And then the gravimetric water content of each sample was measured by oven drying the samples, as shown in Figure 10c. The time required for equilibrium depended on the PAAS ratio and the suction applied to the sample. For this study, the equilibrium time ranged from one day for the PAAS sample with 4 MPa suction to 7 days for the clay sample with 50 kPa suction. Figure 11 shows the soil samples inside solutions on top of magnetic stirrers for 6 different suctions at the same time.

## 3. Prediction Model

Since it is very time consuming to perform suction tests, developing a model to predict the suction potential of PAAS–soil mixtures at any PAAS dosage at a specific water content would be highly beneficial for engineering practice. To predict the SWCCs of PAAS–soil mixtures at various PAAS dosages, five models were developed and evaluated independently in this study, including one mechanistic model and four non-mechanistic models.

The models were developed based on the experimental suction potential data obtained for soil, PAAS, soil + 0.5%PAAS, and soil + 2%PAAS. To evaluate the accuracy of the models, the predicted water contents for soil + 1%PAAS were compared with the actual water content obtained from experiments in this study. This comparison could help determine the variation between prediction and measurements, which was used to evaluate the quality of the prediction models developed by these five methods. This section thoroughly discusses the five methods that were used to develop prediction models.

### 3.1. The Mechanistic Ratio Method

In this method, the obtained SWCCs for both the PAAS and soil were employed to forecast the SWCC of PAAS–soil mixtures with varying SAP proportions. The water content of the PAAS–soil mixture at any given suction level was first calculated under the assumption that the respective contributions of soil and PAAS to the overall mixture’s water content were proportional to their respective mass ratios, and the PAAS and soil could be assumed to behave independently. This model was referred to as the “ratio model”. However, some studies indicated that the capacity of PAAS to absorb water was reduced due to the presence of soil [31,32,33]. Such a reduction could be due to the confining pressure of the soil, or it could be a consequence of the soil’s salinity and the presence of ions in the soil. To account for the reduction, a correction factor for PAAS absorbency was adopted in the ratio model in this study. In this alternative method, referred to as “effective SWCC model”, the water content is calculated based on the PAAS ratio with a correction factor for each PAAS–soil mixture according to Equation (5).

(5) $$\omega_{m}^{i}=\omega_{s}^{i}\left(1-R\right)+F\times \omega_{PAAS}^{i}\times R$$

where $\omega_{m}^{i}$ is the water content of the PAAS–soil mixture at the ith suction; $\omega_{s}^{i}$ is the water content of soil at the same ith suction; $\omega_{PAAS}^{i}$ is the water content of PAAS at the same ith suction; and F is the correction factor for PAAS’s absorbency when mixed with soil.

The correction factor was calculated over the suction range. In this process, the variable $\omega_{m}^{i}$ in Equation (5) was replaced with experimental data from soil samples mixed with varying concentrations of PAAS (0.5%, 1%, and 2%). The correction factors for each PAAS ratio were then averaged. The resulting average correction factor (F) can be used to adjust the SWCC for the PAAS, accounting for the influence of soil.

### 3.2. Polynomial Interpolation

Polynomial interpolation is a non-mechanistic method used to approximate a cluster of data by a polynomial equation. The Lagrange interpolating polynomial uses Lagrange basis polynomials to create a polynomial of degree n − 1, where n is the number of data points, that passes through a set of given data points. The Lagrange polynomial formula is given by Equations (6) and (7).

(6) $$P\left(x\right)=\sum_{j=1}^{n}P_{j}\left(x\right)$$

(7) $$P_{j}\left(x\right)=y_{j}\prod_{k=1}^{n}\frac{x-x_{k}}{x_{j}-x_{k}}$$

We compute the equilibrium water content at every suction level for the soil mixed with 1% PAAS, drawing on the data from four other SWCC of distinct PAAS–soil combinations. Hence, in Equations (6) and (7), ‘n’ is 4, leading to a third-degree polynomial, given that we obtained four datasets from our experiments. Polynomial interpolation using Lagrange interpolating polynomial has several advantages, such as simplicity, computational efficiency, and accuracy in approximating functions with a small number of data points. It is also a flexible method that can be applied to various types of data sets, including irregularly spaced data points [63]. However, there are also some disadvantages to the method, such as the potential for oscillations between data points and the need for higher-order polynomials to accurately approximate highly nonlinear functions. Additionally, the method may not be suitable for large data sets, as it can become computationally intensive. Despite these limitations, the Lagrange interpolating polynomial is a useful tool for many engineering and scientific applications, such as signal processing, numerical integration, and curve fitting [63].

### 3.3. K-Nearest Neighbors (KNN)

**Figure 12:**
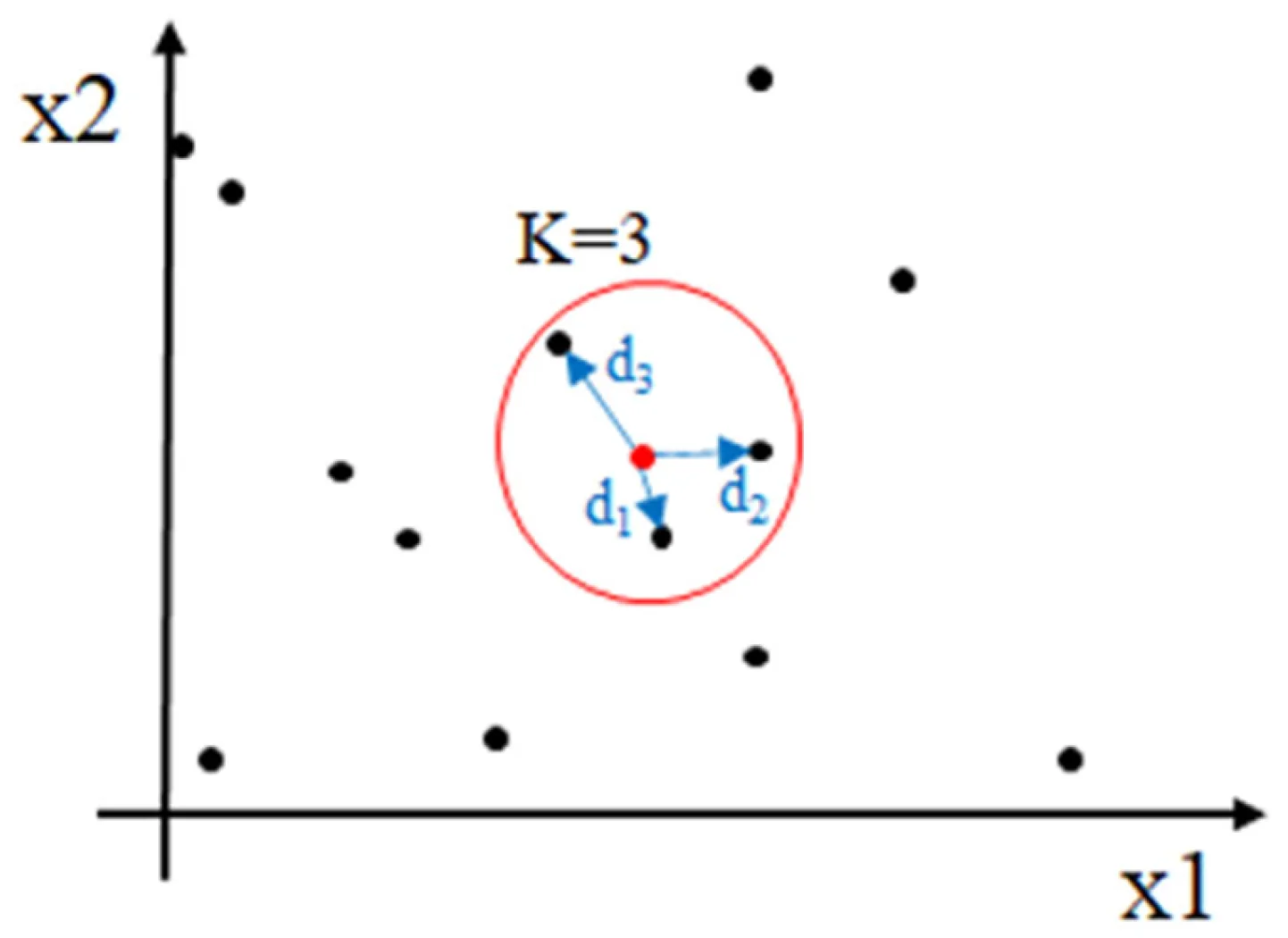
Illustration of KNN algorithm with three neighbors (red—target data point, black—neighboring data points).

The KNN algorithm is another non-mechanistic method that has been a popular topic in machine learning. It works by comparing a query point to the k closest points in a training dataset to determine its class or estimate its value [64]. Figure 12 shows how the KNN algorithm works with a specific value of k (k = 3).

The algorithm is simple, easy to implement, and does not require any training phase [65,66]. KNN uses all the available examples in the dataset to estimate the output of a new case based on the weighted average of its neighbor value. The weight and neighbors are identified based on a similarity measure [67]. The similarity measure chosen for this study was the Euclidean distance given in Equation (8).

(8) $$d\left(x,y\right)=\left\|x-y\right\|=\sqrt{\sum_{i=1}^{n}{\left(x_{i}-y_{i}\right)}^{2}}$$

Euclidean distance is a measure to determine which datapoints are closest to a given query point. The number of datapoints being checked as neighbors (K) should be defined and tuned based on each dataset [64]. Based on the experimental dataset provided in this study, unscaled data were selected to feed the KNN model. The number of neighbors considered (K) was selected to be three and the weight of neighbors was selected to be one. Thus, the predicted water content was the average water contents of the three closest neighbors. KNN has a relatively low computational time and can handle nonlinear decision boundaries. However, KNN can be sensitive to the choice of the number of neighbors (K) and the distance metric used to measure the similarity between data points [64].

### 3.4. Polynomial Regression

Polynomial regression is a non-mechanistic machine learning algorithm used to make predictions. It is a special case of multiple linear regression in which the simple linear regression is extended by constructing polynomial features from the variables [68]. These features can be combined in a polynomial relationship of nth degree within a linear model to fit the data. The polynomial is chosen as a hypothesis function and the performance of the model is evaluated by the cost function [69]. Gradient descent is used to find the coefficients of the hypothesis function in order to minimize the cost function. Based on the suction potential dataset, the main variables of our model were SAP ratio and suction values. Based on these two variables, other features were created and the polynomial model was selected using trial and error based on the *p*-values of each created feature and the mean squared error of the model. A *p*-value is a measure of how likely the null hypothesis is true and that any particular feature is not significant in predicting the output. Thus, a feature with a low *p*-value is probably significant in predicting the output of a larger population [69]. The selected features of the polynomial and their *p*-values are shown in Table 2. The polynomial is shown in Equation (9).

(9) $$\omega =-0.717+114.39R+4.75h-603.81\left(R\times h\right)+510.42\left(R^{2}\times h^{2}\right)+286.25\left(\frac{R}{h^{2}}\right)$$

where ω is the water content, R is the PAAS ratio, and h is the suction value.

### 3.5. Deep Neural Network (DNN)

Deep neural networks are a class of artificial neural networks that are capable of learning complex patterns and relationships in data [70]. They are composed of multiple layers of interconnected nodes or neurons and each layer transforms input data into a higher-level representation that captures increasingly abstract features [66]. Deep neural networks are trained using large amounts of labeled data and a technique called backpropagation, which adjusts the weights of the network to minimize the error between its predictions and the true labels. DNN has the advantage of predicting highly nonlinear relations by creating new features at various levels of complexity in each layer [71,72].

**Figure 13:**
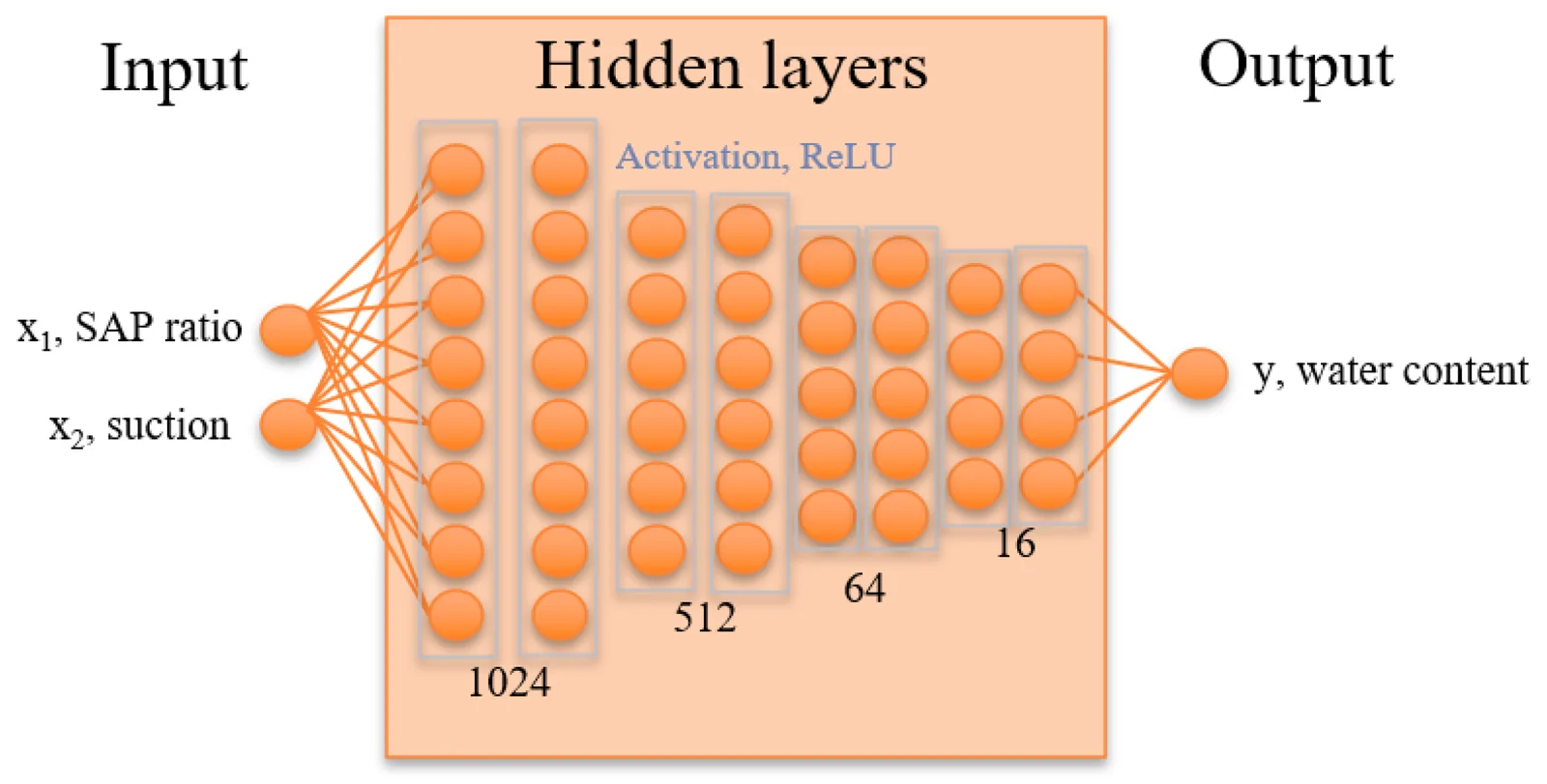
Schematic of the DNN architecture.

The number of layers and neurons in each layer, as well as the type of activation functions used, are important architectural choices that can greatly impact the performance of the network [66]. Common types of layers used in DNNs include fully connected layers, convolutional layers, and recurrent layers, each designed to handle different types of data and tasks [70,73]. The architecture of DNN is often designed through a process of trial and error and is heavily dependent on the specific problem and dataset being analyzed. For this study, we created a DNN consisted of eight layers using Keras in TensorFlow [74]. The first two layers contained 1024 neurons, the second two layers contained 512 neurons, the fifth and sixth layers contained 64 neurons, the seventh layer contained 16 neurons, and the last layer represented the output with one neuron. The architecture of the DNN is shown in Figure 13. ReLU (Rectified Linear Units) activation function was used in each layer as a nonlinear function except for the final output layer to avoid the problem of vanishing gradients during backpropagation [75]. ReLU has several advantages over other activation functions, including its computational efficiency and ability to alleviate the vanishing gradient problem [76,77].

For updating the weights of a neural network during training, Adam (adaptive moment estimation) was used as the optimizer that is a combination of the ‘gradient descent with momentum’ algorithm and the Root Mean Square Propagation (RMSP) algorithm. Adam was able to handle sparse gradients and noisy data, and has been shown to perform well on a wide range of deep learning tasks [78]. Adam maintained a set of exponentially decaying average of past gradients and past squared gradients, known as momentum and variance respectively, for each weight parameter in the network. These estimates were then used to update the weight parameters with a learning rate that was adaptively scaled for each parameter.

The batch size hyperparameter that controlled the speed and stability of the learning process was selected to be 32 based on trial and error. The batch size is a hyperparameter that specifies the number of training examples used in each iteration of the training process of a deep neural network. Instead of updating the network weights after every individual training example, the training data were divided into batches of fixed size, and the weights were updated based on the average gradient of the loss function computed over the batch. Choosing an appropriate batch size was important for balancing the tradeoff between computational efficiency and the quality of the learned model [79]. A small batch size could lead to more frequent weight updates and faster convergence, but might result in noisy gradients that could cause the training process to converge to a suboptimal solution. On the other hand, a large batch size could provide more accurate gradient estimates, but could be computationally expensive and required more memory [80].

In DNNs, the number of epochs refers to the number of times the entire training dataset is presented to the network during the training process. Each epoch consists of multiple iterations, where the network processes one batch of data at a time and updates their weights based on the errors between predictions and the true labels. The number of training epochs is a critical hyperparameter that must be selected carefully. An insufficient number of epochs can lead to underfitting, where the model fails to adequately capture the underlying patterns in the data, whereas an excessive number of epochs can result in overfitting, causing the model to memorize the training data and perform poorly on unseen data [81]. The number of epochs for this study was optimized and chosen to be 400 to run based on the loss of the training and test set.

## 4. Results and Discussions

### 4.1. Established SWCC

**Figure 14:**
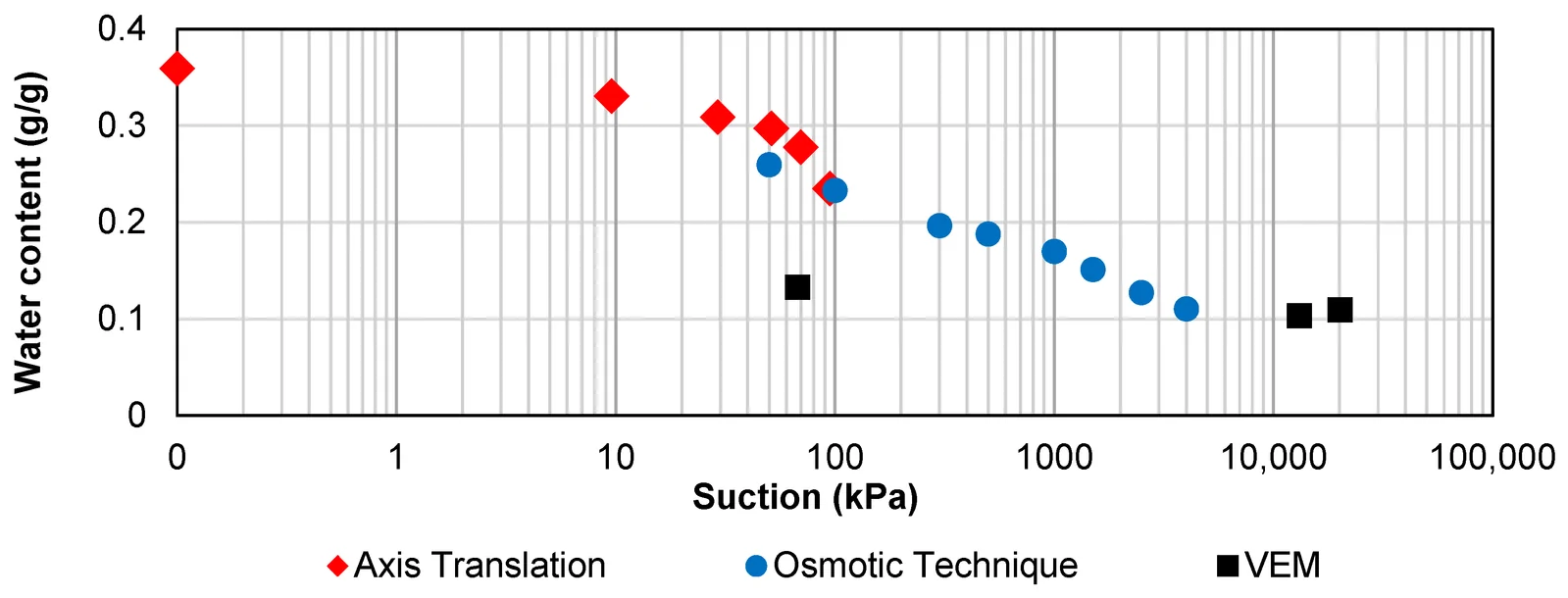
SWCC for soil with three different methods of axis translation, osmotic technique, and vapor equilibrium technique.

Three different soil suction measuring methods, i.e., axis translation, osmotic and vapor equilibrium, were used to measure the low, medium and high suctions, respectively. The resulting water contents obtained at any suction value with each method are put together to plot the graph of SWCC for the soil only without PAAS as shown in Figure 14.

The data points collected with each of these three methods match and supplement each other well. The overlap in suction range of 50 kPa to 100 kPa between the two methods shows that the axis translation method and osmotic technique provide good agreement for the obtained water content. The obtained water content for these two methods had less than 15% difference for the 50 kPa suction and nearly the same water content was obtained for the 100 kPa suction. However, the difference between the results of the axis translation method and vapor equilibrium method for the suction of 70 kPa is more than 100% which shows the inefficiency of vapor equilibrium technique for lower suctions, which has been pointed out by many studies [82,83,84]. Based on the results of SWCC for soil with different methods and their overlapping suction range, it is decided that the axis translation method using the Tempe cell would be used for suctions of 0.1, 10, and 30 kPa, the osmotic technique is best for suctions in range of 50–4000 kPa, and the vapor equilibrium technique should be used for suctions beyond 10 MPa.

**Figure 15:**
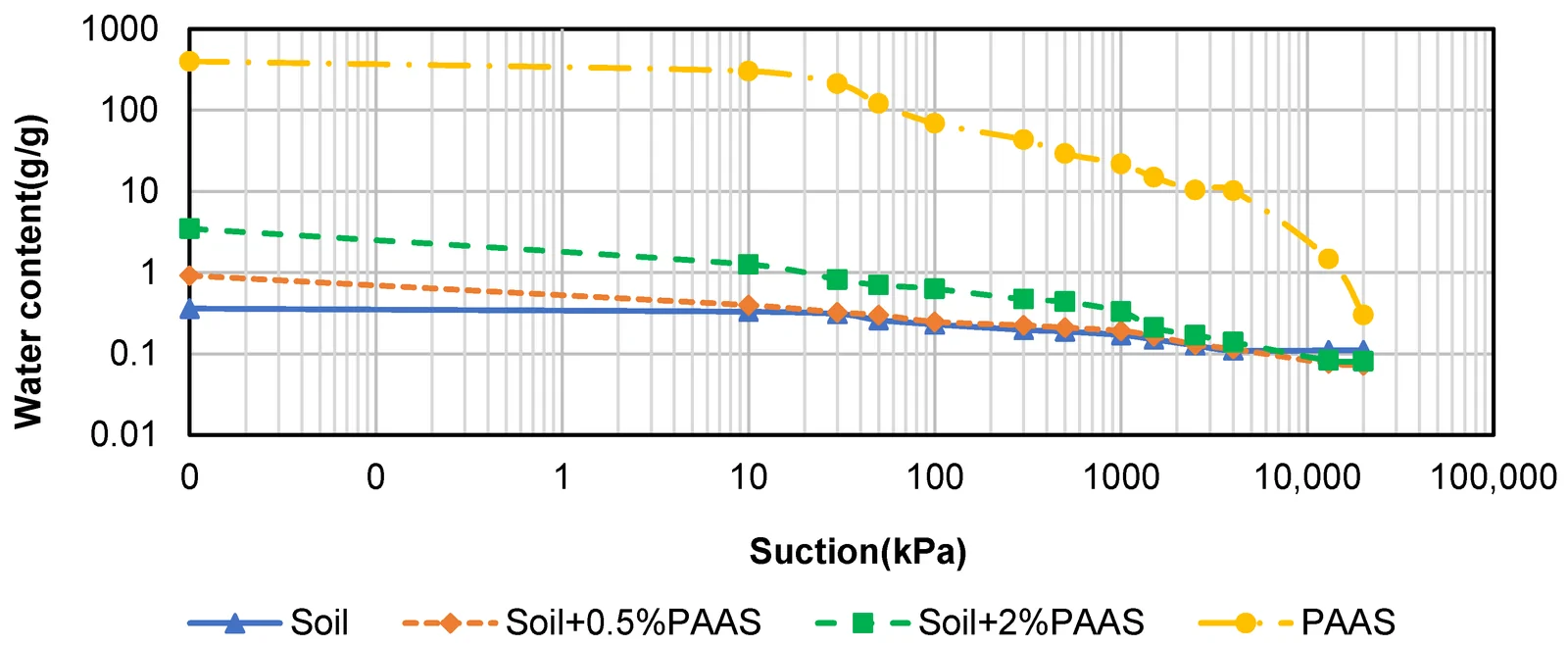
SWCC for soil, PAAS and different PAAS–soil mixtures.

The resulting SWCCs for PAAS and the PAAS–soil mixtures are shown in Figure 15 and their water content data for any suction value are listed in Table 3.

As shown in Figure 15, the water holding capacity of PAAS–soil mixtures increases significantly with the increase in the PAAS ratio for suctions up to 6000 kPa. In wet conditions represented by the point with 0.01 kPa of suction, clay with 0.5% PAAS is able to hold more than twice the water than soil itself. Addition of 2% PAAS to clay improves its water holding capacity up to 10 times in wet conditions. However, addition of PAAS seems to slightly decrease the water holding capacity of clay for suctions higher than 6000 kPa. PAAS can hold water up to a thousand times more than clay under moist conditions. However, when suction is greater than 1000 kPa, a sharper gradient is observed in the PAAS’s SWCC, as compared to the gentle curve of clay, which suggests that PAAS discharges water more readily. A shift in suction from 0.01 to 20,000 kPa decreases the water content in soil by 70%, whereas for PAAS, the reduction amounts to nearly 100%. This signifies that PAAS is more susceptible to water loss under equivalent suction changes compared to soil. This confirms our hypothesis that PAAS can help to absorb the excess water in wet conditions and to release the water to dry soil in drier conditions.

**Figure 16:**
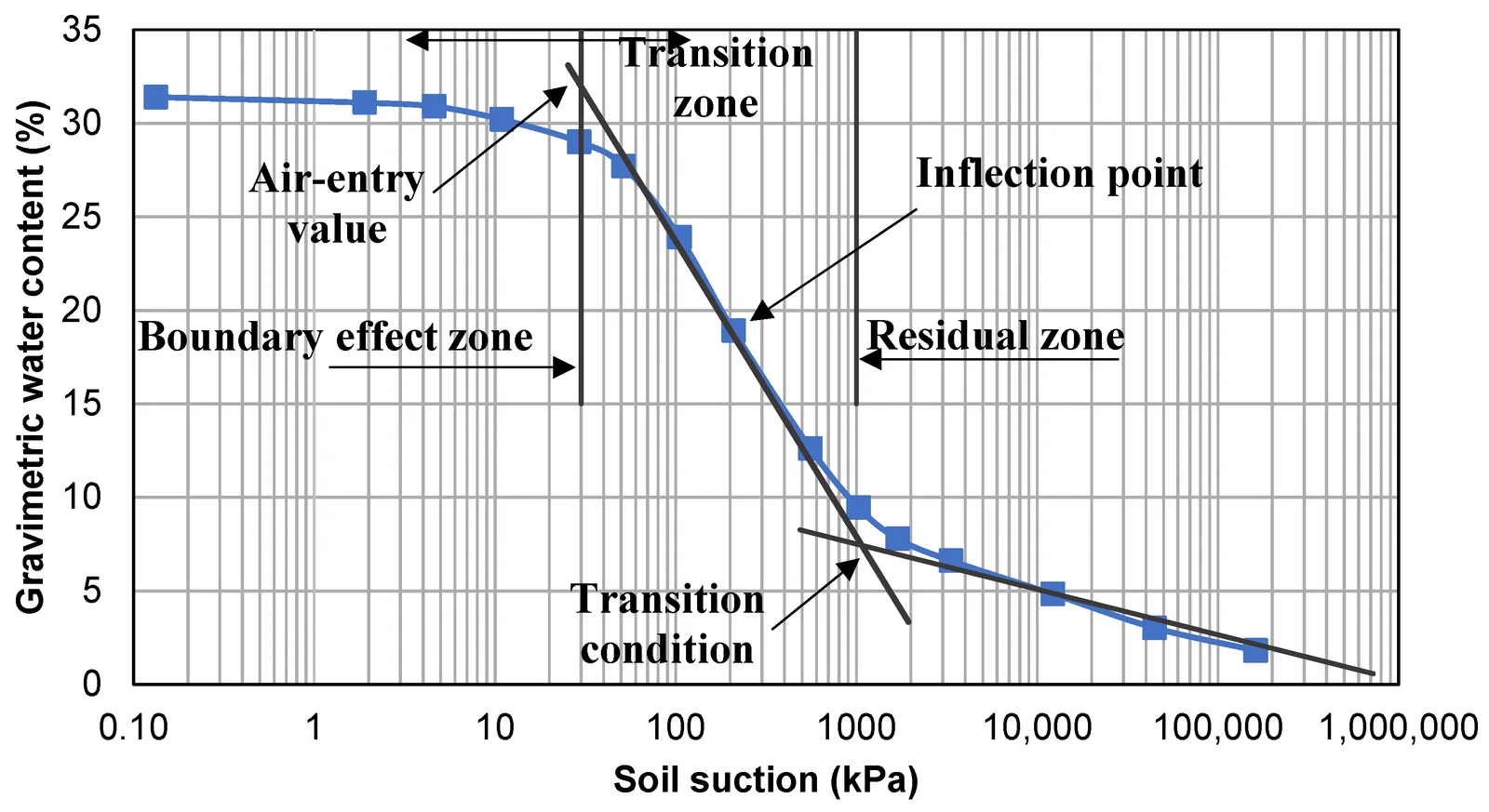
Illustration of the in situ zones of desaturation defined by a soil–water characteristic curve; redrawn from [85].

For a closer analysis of the SWCC, the desaturation of soil is typically divided into three zones: the boundary effect zone, the transition zone, and the residual zone as illustrated in Figure 16. These zones are determined by two distinct points on the SWCC: the soil’s air-entry value and residual conditions [85].

The air-entry value of soil represents the matric suction, at which air begins to displace water in the soil’s largest pores. The residual water content, on the other hand, refers to the amount of water in the soil that requires a greater change in suction to remove any additional water. In other words, there is a notable shift in the rate at which water can be extracted from the soil. The gradient of a line connecting the air-entry value and the residual point (i.e., the slope at the inflection point) is a measure of the amount of water extracted from the soil as suction increases beyond the air-entry value. The slope of the SWCC, on the other hand, indicates how quickly water storage in the soil changes with respect to soil suction [44].

**Figure 17:**
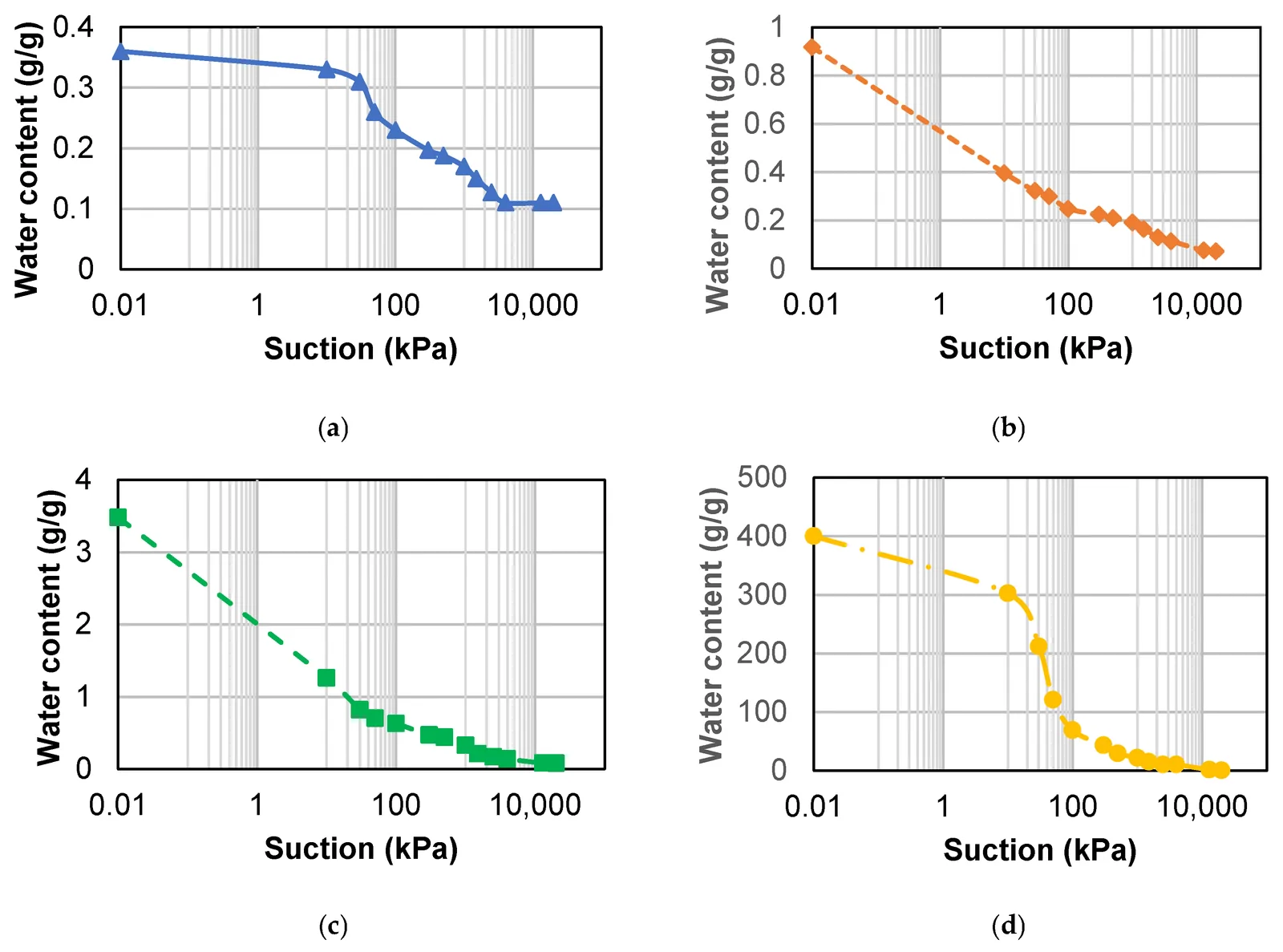
Comparing the SWCC of soil, PAAS, and PAAS–soil mixtures: (**a**) soil, (**b**) soil + 0.5%PAAS, (**c**) soil + 2%PAAS, and (**d**) PAAS.

The SWCCs of soil and PAAS (shown in Figure 17a and Figure 17d, respectively) clearly show a similar pattern as Figure 16. Even though the water-absorbing mechanism of PAAS involves the substitution of the water molecule’s hydrogen ions with positively charged sodium ions, which are bound to the polymer’s negatively charged carbonyl groups, it seems that the interaction between water and air in soil or PAAS is similar. Therefore, SWCC of PAAS shows a similar pattern as that of soil.

The SWCC of PAAS–soil mixtures exhibit neither a transition zone nor a boundary effect zone. Moreover, at saturation, the water content for the soil + 2%PAAS mixture peaks at 3.48 g/g, a value that represents merely 1% of the PAAS’s absorbency at saturation when it is isolated. As per the SWCC of the standalone PAAS, a water content of 3.48 g/g suggests that the PAAS is still in its residual zone and has yet to exploit much of its absorption capacity. This observation could account for the absence of a transition zone and a boundary effect zone in the SWCC of PAAS–soil mixtures. In addition, the soil has already achieved its maximum water content when the mixture is saturated. Hence, it can be inferred that when mixed with soil, the absorbency of PAAS is restricted to its residual zone, and the PAAS fails to achieve its optimal swelling, even when the mixtures reach a state of complete saturation. This could be due to the confinement of soil structure which prevents the swelling of PAAS grains or it could be linked to the presence of ions and the impact of soil salinity on diminishing the PAAS’s absorbency.

### 4.2. SWCC Prediction

**Figure 18:**
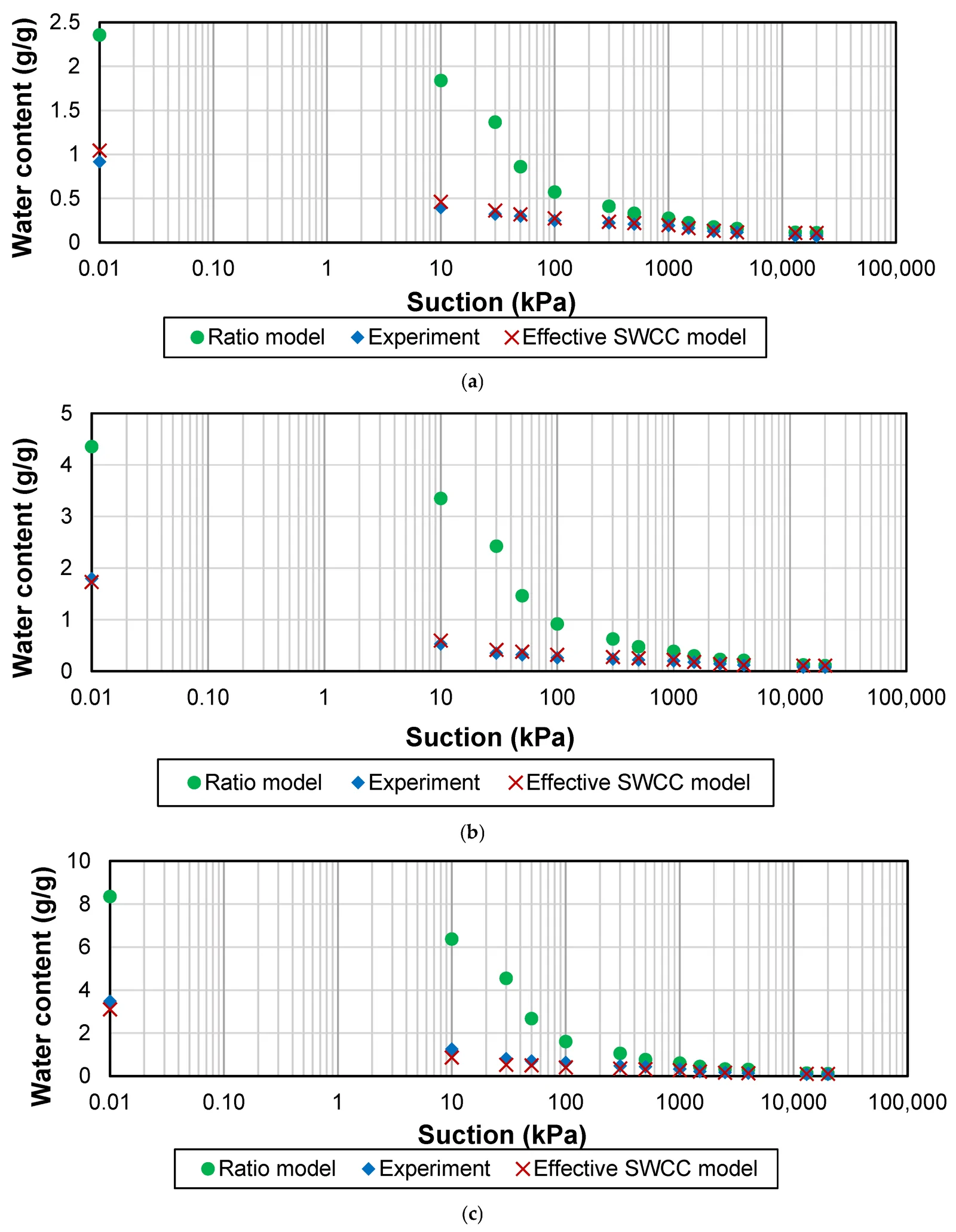
Predicted SWCC with simple ratio model compared with the established SWCC from experiments for PAAS–soil mixtures: (**a**) soil + 0.5%PAAS, (**b**) soil + 0.5%PAAS, and (**c**) soil + 0.5%PAAS.

The accuracy of the simple ratio model in predicting the water content was evaluated by making predictions for PAAS–soil mixtures with 0.5%, 1%, and 2% PAAS ratios and comparing the predictions with the experiment data. Figure 18 compares the SWCCs from experiments with the predicted SWCC from the ratio model and the effective SWCC model.

The ratio model only took into account the effect of PAAS and soil proportions in a mixture, which yields a reasonable match with the measured suction in the residual zone, i.e., high suction range (500–20,000 kPa). However, it fails to accurately represent the SWCC for low suctions. The considerable discrepancy between the outcomes of this model and the experimental data for lower suctions underscore that the behavior of PAAS and soil is interactive and not independent. Hence, it is imperative to incorporate a correction factor when developing a predictive model.

The interval of suction where the ratio model predictions are reliable ranges from 500 to 20,000 kPa, corresponding to the residual zone of the PAAS’s SWCC. This may be attributed to the observation that, upon being combined with soil, the swelling and absorbency of PAAS never attains its maximal swelling at suctions. That means the suction potential of PAAS was greatly suppressed by the suction potential of soil. Such a finding can be supported by the data presented in Figure 17a,d, which show that under very dry conditions (content is nearly zero) the suction in soil and PAAS are comparable. However, as the water content increases, the suction of soil vanishes much more rapidly than that of PAAS. The effective SWCC model aligns well with the experimental data, validating that adjustments for the PAAS’s absorbency are necessary to consider the impedance of PAAS suction under the impact of soil when water content is low.

**Figure 19:**
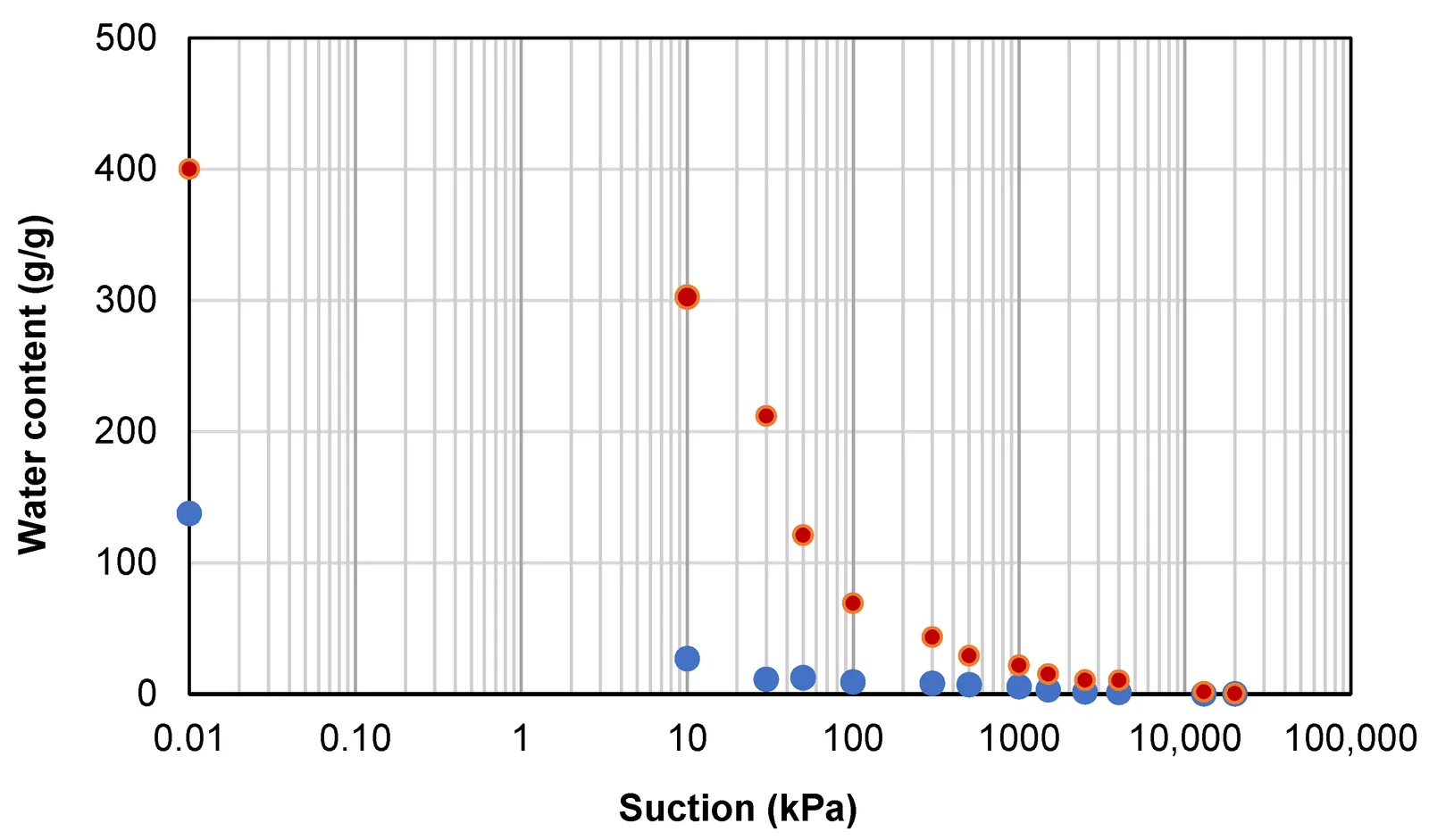
Comparison between the calculated effective SWCC of PAAS when mixed with soil (blue) and the original SWCC of PAAS (red).

To manifest the impact of soil on PAAS suction and absorbency, Figure 19 compares the SWCC of PAAS with and without soil presence. To make the comparison easy to understand for both wet and dry zones, log scales are used for both vertical and horizonal axes. The SWCC of PAAS is isolated from the SWCC of soil–PAAS mixture by applying a correction factor, as shown in the second term in Equation (5). Comparing the effective SWCC of PAAS with the original SWCC, the peak absorbency of PAAS when confined by soil is observed at 140 g/g when the mixtures attain saturation. This suggests that when integrated with soil, PAAS’s swelling is limited to approximately 35% of its 400 g/g potential at saturation due to the restrictive nature of the soil structure. A correction factor of 0.35 compares well with the results of Misiewicz et al. [36] that had a PAAS load factor of 0.34 at saturation for a 0.3% PAAS coarse sand mixture.

In addition to the above-discussed simple prediction model based on mixing ratios, this study explores other numerical approaches, including machine learning, to establish prediction models based on SWCC for soil + 0.5%PAAS and soil + 2%PAAS. The SWCC for soil + 1% is used to validate the prediction model. Incorporating the acquired experimental data on the SWCCs of soil + 0.5%PAAS and soil + 2%PAAS into the models implicitly accounts for the influence of soil’s confinement on the absorbency of PAAS that takes place when PAAS is blended with soil. This consideration is necessary since such an effect is inherent in the experimental data for the SWCC of the mixtures.

**Figure 20:**
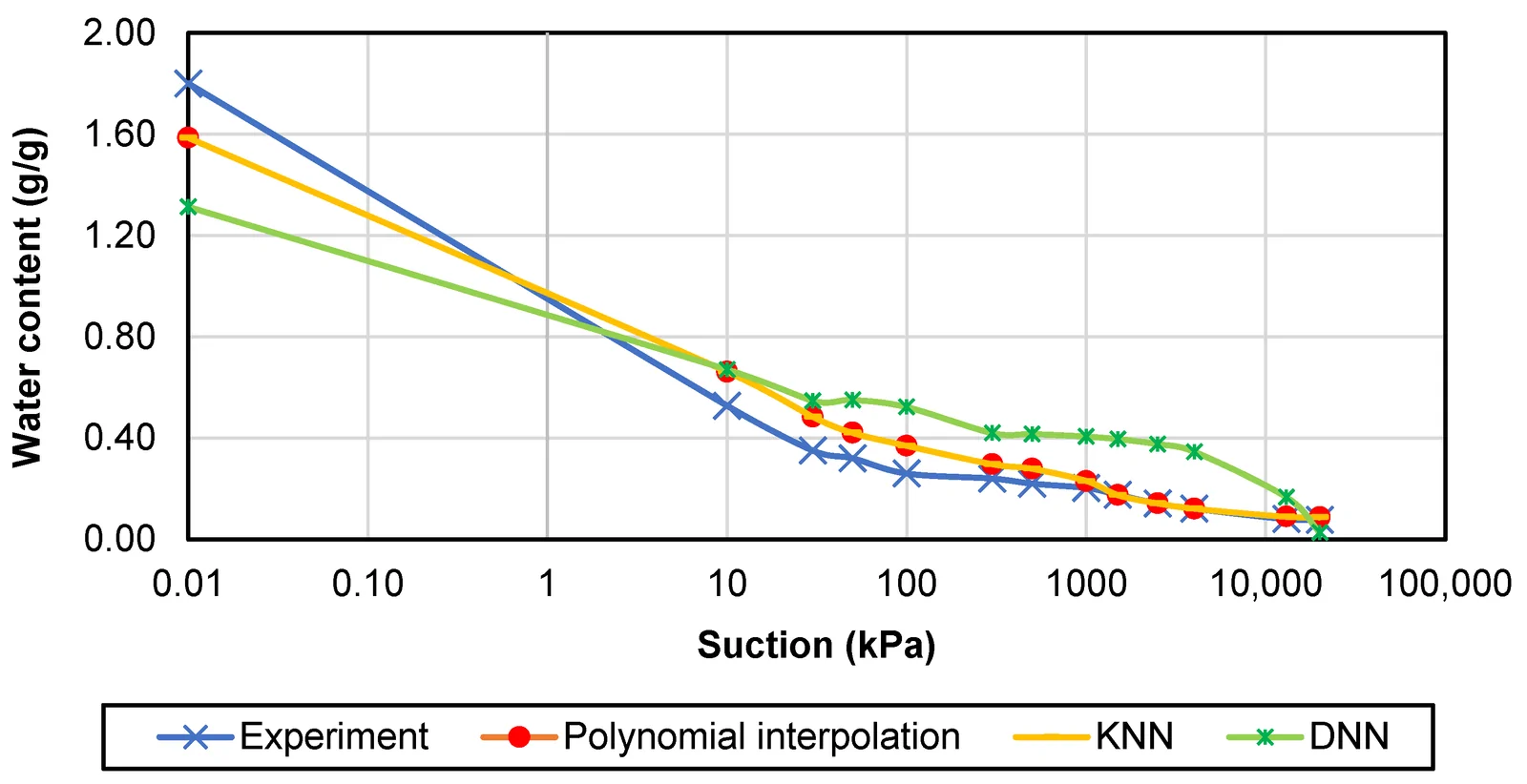
Comparison between the predicted SWCC of soil + 1%PAAS and the experiment (note: KNN and polynomial data have insignificant difference).

Figure 20 displays the predictions for soil + 1%PAAS obtained by various methods, along with the data obtained in experiments. While polynomial interpolation, KNN, and DNN are found to produce accurate and acceptable outcomes among various prediction methods, the predicted water contents derived from the polynomial regression model had the lowest accuracy despite numerous attempts to construct various features using trial and error methods. Therefore, the resulting predictions of the polynomial regression model are not presented in this study. In this study, mean squared error (MSE) and R-squared are utilized as two widely adopted metrics for evaluating the precision of the model’s predictions. According to MSE and R-squared values, polynomial interpolation and KNN provide the most accurate predictions for the water contents of PAAS–soil mixtures; it is proposed that these models can be utilized to establish SWCCs for soils combined with PAAS. This recommendation assumes that the SWCC of the soil, PAAS, and at least two other PAAS–soil mixtures are used as input data. Such a strategy could facilitate engineers in conveniently leveraging their existing SWCC data to generate additional SWCCs. Even though the trained DNN model shows acceptable accuracy in predicting the water content of soil + 1%PAAS, it is not suggested for further usage as it is more suitable for application with large dataset(s).

## 5. Conclusions

The ability of PAAS in retaining large volumes of water and also releasing it in dry conditions makes it suitable as an additive to soil for geotechnical applications. It has great potential to become a new soil stabilizer for various infrastructure applications such as slopes, earthen structures, and foundations. In order to evaluate the efficiency of PAAS in improving engineering properties of clay, SWCC of soil and different PAAS–soil mixtures are established with three different methods. Comparing the obtained SWCC for each mixture with soil and PAAS curves show that addition of PAAS increases the suction potential of mixtures significantly for suctions up to 6000 kPa. For higher suctions, however, PAAS slightly decreases the water holding capacity. This could indicate the ability of PAAS in releasing the water back to soil in drier conditions since clay has a higher suction potential than the mixtures in drier conditions. The absence of a transition zone and a boundary effect zone in the SWCC of PAAS–soil mixtures suggests that the swelling of PAAS is limited when combined with soil due to the restrictive nature of the soil structure and also the presence of ions and soil salinity. As a result, the water absorption capacity of PAAS, when mixed with soil, is much less than suggested by its SWCC by itself. This finding provides basic guidance if PAAS is used to prevent moisture fluctuations in the soil for engineering purposes.

A basic ratio model, which is based on the derived SWCC of soil and PAAS, provides a fairly accurate match with the established SWCC for higher suctions in the residual zone. However, it is unable to accurately depict the SWCC for lower suctions. This further supports the observation that PAAS’s ability to absorb water is confined to the residual zone within its SWCC when mixed with soil, and its swelling capacity is limited to approximately 35% of its potential due to the constraining attributes of the soil structure at saturation.

In practice, SWCC is difficult to establish for every PAAS–soil mix due to the time-consuming experimental methods, especially for higher suction ranges. Hence, various techniques are employed to forecast the water content of PAAS–soil mixtures at any given suction level. Among predictive models that leverage the experimentally derived SWCC of soil + 0.5%PAAS and soil + 2%PAAS as inputs, the Lagrange interpolating polynomial is the most accurate model in predicting the water content of PAAS–soil mixtures. But there are a higher computation effort and a higher error rate with this mathematical method. The K-nearest-neighbors (KNN) is a simple and quick method with an accuracy nearly comparable with that of the interpolating polynomial method.

While PAAS has been extensively applied as a soil conditioner in agriculture, its potential applications in civil engineering have received limited attention. Therefore, additional studies are required to characterize the performance of PAAS under diverse field conditions, including variations in pH, temperature, and soil gradation. Furthermore, future research should not only advance the understanding of soil–PAAS–water interactions but also examine the effects of PAAS on critical engineering properties, including shear strength, compressibility, and failure mechanisms. Finaly, the potential for biodegradability of PAAS should also be investigated when considering longer-term applications.

## Figures and Tables

**Table 1:** Soil properties.

Material	Max. Dry Unit Weight (kN/m^3^)	LL (%)	PL (%)	SL (%)	PI (%)
Soil	17.5	34	15	12	19

Note: LL—liquid limit, PL—plastic limit, SL—shrink limit, PI—plasticity index.

**Table 2:** Features used in the polynomial regression model and their *p*-values.

Features	*p*-Values
PAAS ratio (R)	0.0000
Suction (h)	0.8241
PAAS ratio*suction (R × h)	0.0005
PAAS ratio^2^*suction^2^ (R^2^ × h^2^)	0.0035
PAAS ratio/suction^2^ (R/h^2^)	0.0000

**Table 3:** The water content of different PAAS–soil mixtures versus suction value.

Suction (kPa)	Water Content (g/g)			
	Soil	Soil + 0.5%PAAS	Soil + 2%PAAS	PAAS
0.01	0.36	0.92	3.48	400.00
10	0.33	0.40	1.26	302.40
30	0.31	0.32	0.82	211.84
50	0.26	0.30	0.70	120.88
100	0.23	0.25	0.63	69.00
300	0.20	0.22	0.47	43.25
500	0.19	0.21	0.44	29.10
1000	0.17	0.19	0.33	21.70
1500	0.15	0.17	0.21	14.95
2500	0.13	0.13	0.17	10.34
4000	0.11	0.11	0.14	10.16
13,000	0.11	0.08	0.08	1.47
20,000	0.11	0.07	0.08	0.30

## Data Availability

The original contributions presented in this study are included in the article. Further inquiries can be directed to the corresponding author.
